# Ultrasensitive and specific fluorescence detection of a cancer biomarker *via* nanomolar binding to a guanidinium-modified calixarene†
†Electronic supplementary information (ESI) available: Experimental details, additional characterisation data. See DOI: 10.1039/c7sc04989g


**DOI:** 10.1039/c7sc04989g

**Published:** 2018-01-09

**Authors:** Zhe Zheng, Wen-Chao Geng, Jie Gao, Yu-Ying Wang, Hongwei Sun, Dong-Sheng Guo

**Affiliations:** a College of Chemistry , State Key Laboratory of Elemento-Organic Chemistry , Key Laboratory of Functional Polymer Materials , Ministry of Education , Nankai University , Tianjin 300071 , China . Email: dshguo@nankai.edu.cn; b Collaborative Innovation Center of Chemical Science and Engineering , Nankai University , Tianjin 300071 , China

## Abstract

We designed a water-soluble guanidinium-modified calix[5]arene to target lysophosphatidic acid (LPA), an ideal biomarker for early diagnosis of ovarian and other gynecologic cancers, achieving binding on the nanomolar level. An indicator displacement assay, coupled with differential sensing, enabled ultrasensitive and specific detection of LPA. Moreover, we show that using a calibration line, the LPA concentration in untreated serum can be quantified in the biologically relevant low μM range with a detection limit of 1.7 μM. The reported approach is feasible for diagnosing ovarian and other gynecologic cancers, particularly at their early stages.

## Abstract 2

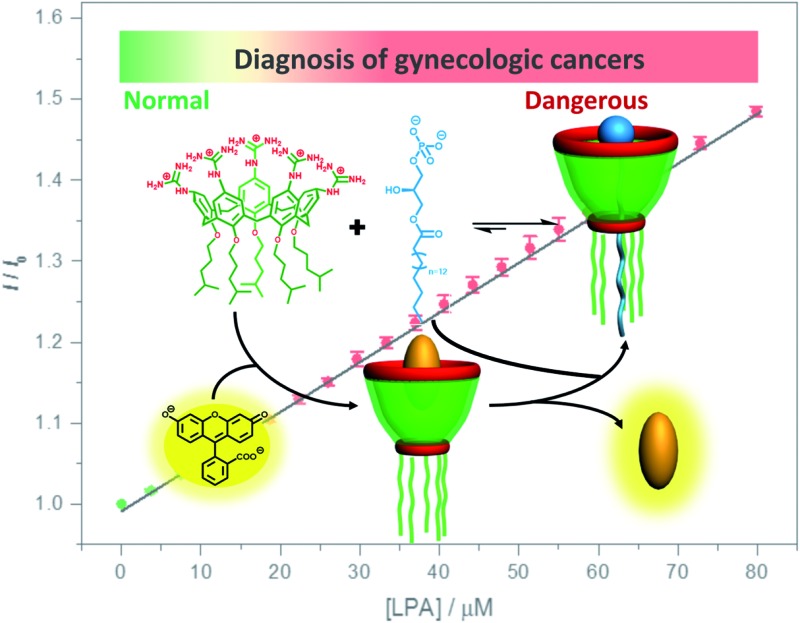
A well-designed macrocycle affords nanomolar binding to a cancer biomarker lysophosphatidic acid, showing potential application in the diagnosis of gynecologic cancers.

## Introduction

Quantitative detection of cancer biomarkers, particularly those non-invasive in plasma, is of great importance for early diagnosis, which facilitates effective treatment and improves the survival rate of cancer patients.^1^ Lysophosphatidic acid (LPA), a type of bioactive phospholipid, is an ideal biomarker for the early detection of ovarian and other gynecologic cancers.^2^ The LPA concentrations in healthy human plasma are approximately 0.1–6.3 μM and the danger levels of LPA for gynecologic cancers are indicated by concentrations of the order of 63.2 μM.^3^ The routine diagnostic testing of the plasma LPA level is limited in present detection techniques, such as tandem mass spectroscopy, capillary electrophoresis and radio-enzymatic assays,^4^ which need sophisticated devices and complicated procedures. Optical methods (*via* fluorescence or colorimetric changes) represent powerful sensing modalities due to their low cost, ease of use and high sensitivity.^5^ To date, there have been several examples, in which LPA was detected by optical methods, but they generally were hindered by low sensitivity and/or poor specificity.^6^ Consequently, quantitative detection of plasma LPA by optical methods still requires complicated sample pretreatment to remove most, if not all, the interfering substances.6f,g The key bottleneck is the specific recognition of LPA with strong affinity by artificial receptors. Due to the aforementioned low physiological concentration of LPA, it is highly desirable to design artificial receptors affording extremely strong binding to LPA with exquisite specificity.

Macrocyclic hosts are a family of well-developed artificial receptors with a discrete cavity that is selective for complementary binding to certain guests. With respect to the efficient host–guest interactions between macrocycles and biological substrates in aqueous media, their molecular recognition has gained considerable attention and demonstrated various applications in, but not limited to, the fields of disease diagnosis and therapy, such as sensing of biomarkers,^7^ enhancing solubility and stability of drugs,^8^ regulating protein–protein interactions,^9^ and inhibiting amyloid fibril formation.^10^ Despite these significant achievements, to the best of our knowledge, a macrocycle that affords strong binding and specific detection of LPA has never been reported.

**Scheme 1:**
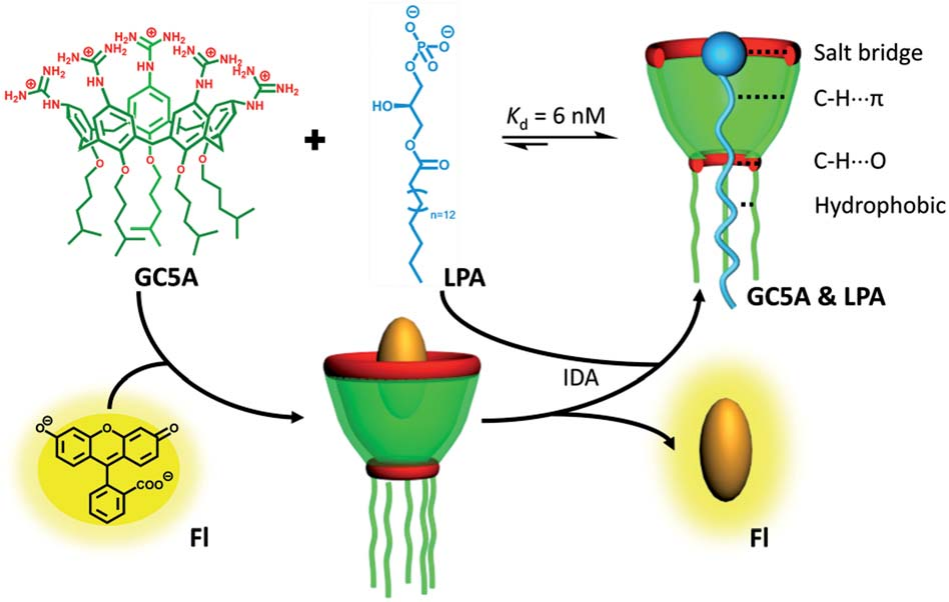
Schematic illustration of the binding between LPA and GC5A and the operating IDA principle of fluorescence “switch-on” sensing of LPA by the GC5A·Fl reporter pair.

As part of our ongoing research, which explores biomedical applications by taking advantage of the molecular recognition of calixarene macrocycles,^11^ we herein designed a water-soluble guanidinium-modified calix[5]arene (GC5A), affording the desired strong binding and ultrasensitive fluorescence detection of LPA *via* an indicator displacement assay (IDA) in aqueous media (Scheme 1). To deal with the presence of interfering substances and the complicated physiological milieu, differential sensing was then introduced to differentiate LPA from all other biologically important species in plasma. In particular, we achieved quantitative detection of LPA in the biologically relevant low μM range in serum without any complicated pre-treatment procedures, demonstrating that this approach has potential for point-of-care testing.

## Results and discussion

**Scheme 2:**
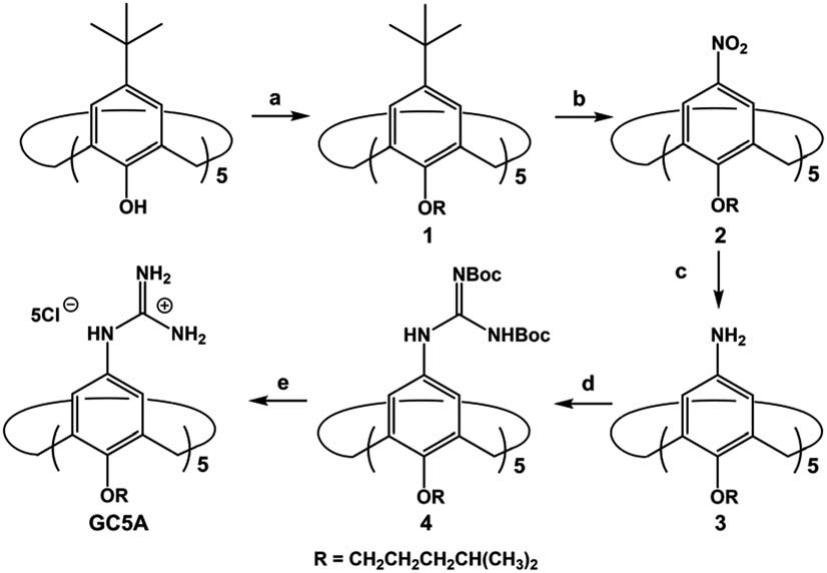
Synthetic route for GC5A. (a) K_2_CO_3_, RBr, CH_3_CN, reflux, 72%; (b) HNO_3_, AcOH, dry CH_2_Cl_2_, r.t., 46%; (c) SnCl_2_·2H_2_O, C_2_H_5_OH/AcOEt (1 : 1, v/v), reflux, 52%; (d) *N*,*N*′-bis-*tert*-butoxycarbonylthiourea, Et_3_N, AgNO_3_, dry CH_2_Cl_2_, r.t., 32%; (e) SnCl_4_, AcOEt, r.t., 65%.

LPA possesses two potential binding sites: one phosphate head and one long-chain fatty-acid tail. Referring to the structural features of LPA, we designed the artificial receptor GC5A. First, calixarenes were employed as the macrocyclic scaffold, which have been described as having “(almost) unlimited possibilities” benefiting from their facial modification.^12^ Second, among the calix[*n*]arene (C*n*A, *n* = 4, 5, 6, 8 generally) family, we screened C5A on account of its size fit. The alkyl chain effectively threads C5A, but not the smaller C4A, while C6A and C8A are relatively larger and have complex conformations.^13^ Third, guanidinium groups were decorated at the upper rim of C5A to donate charge-assisted hydrogen bonds (salt bridge) with the phosphate head of LPA.^14^ Finally, alkyl chains were attached at the lower rim to provide hydrophobic interaction with the tail of LPA besides rigidifying the C5A conformation. Collectively, these design principles led us to prepare the GC5A host shown in Scheme 1, which is expected to show strong binding to LPA *via* the synergistic effect among several interactions (electrostatic, hydrogen bond, C–H···π and hydrophobic). The GC5A was prepared primarily according to the syntheses of reported C4A analogues (Scheme 2).^15^ In brief, the synthesis started from the maternal *p*-tertbutylcalix[5]arene, which was alkylated at the lower rim to obtain **1** with a well-defined cone conformation.^16^ Subsequently, **2** was generated by treating **1** with HNO_3_ and AcOH to substitute all the *tert*-butyl groups with nitro groups through an *ipso*-nitration reaction. Then, **3** was obtained by reduction of nitro to amino groups by SnCl_2_·2H_2_O in ethanol and ethyl acetate. Then, **4** was obtained *via* reaction with di-Boc-protected thiourea units. Removal of the protecting groups by stannic chloride finally afforded the target GC5A receptor.

The GC5A host and the LPA guest are both amphiphilic with critical aggregation concentrations (CACs) of 0.4 and 0.35 mM (Fig. S9†), respectively.6*h* As a result, measurement of the binding affinity between GC5A and LPA should be implemented at concentrations as far below their CACs as possible to avoid any complications from amphiphilic aggregation. Direct NMR and calorimetric titrations were therefore ruled out since they generally require relatively high concentrations. As an alternative approach, fluorescent IDA that could be operated at low μM or even nM concentrations appears to be a desirable choice. IDA, the use of synthetic receptors with competitive binding assays, has been popularized by Anslyn *et al.* as a standard strategy for molecular sensing, complementary to the approach of direct sensing.7b,^17^ We employed IDA to not only determine the binding affinity between GC5A and LPA, but also to concurrently offer the opportunity for fluorescence sensing of LPA.

**Fig. 1:**
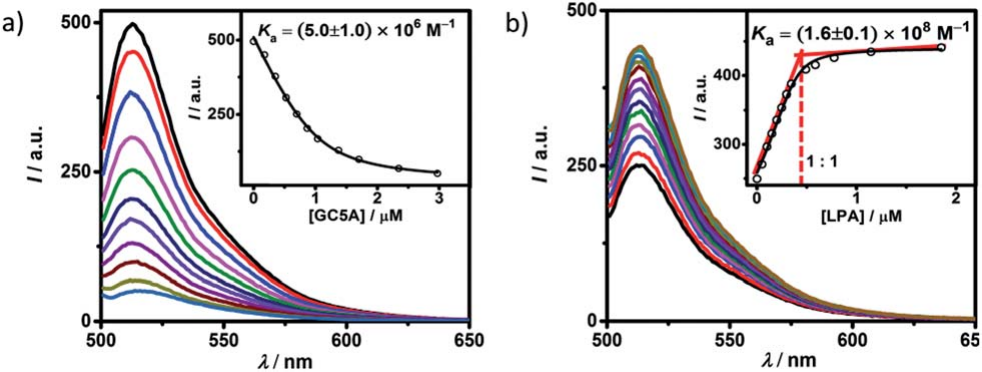
(a) Direct fluorescence titration of Fl (1.0 μM) with GC5A (up to 3.0 μM), *λ*_ex_ = 500 nm. (Inset) the associated titration curve at *λ*_em_ = 513 nm and the fit according to a 1 : 1 binding stoichiometry. (b) Competitive titration of GC5A·Fl (0.4/0.5 μM) with LPA (up to 1.9 μM). (Inset) fit of the titration data to a 1 : 1 competitive binding model. All experiments are in HEPES buffer (10 mM, pH = 7.4) at 25 °C.

Fluorescein (Fl) was screened as the optimal reporter dye, owing to its high brightness, strong binding with GC5A and drastic complexation-induced quenching of fluorescence (Fig. 1a). The binding stoichiometry between GC5A and Fl was determined to be 1 : 1 according to the Job's plot (Fig. S10†). The association constant (*K*_a_), extracted from the fluorescence titration, was fitted as (5.0 ± 1.0) × 10^6^ M^–1^, which was further validated by UV-vis titration (Fig. S11†). In particular, the fluorescence depression upon complexation, *I*_free_/*I*_bound_, is calculated as a factor of 37, which is ideal for the projected IDA application.

The displacement of GC5A·Fl by gradual addition of LPA resulted in regeneration of the intrinsic emission of Fl (Fig. 1b). The data were well fitted by a 1 : 1 competitive binding model, giving a *K*_a_ value of (1.6 ± 0.1) × 10^8^ M^–1^. The 1 : 1 binding stoichiometry was clearly verified by the inflection point at 1 : 1 molar ratio between GC5A and LPA in the competitive titration. To validate the synergistic effect of several interactions on nanomolar binding between GC5A and LPA, we measured the binding of phosphate and 6 : 0 LPA (a shorter analogue) with GC5A, giving *K*_a_ values of (4.6 ± 0.6) × 10^4^ M^–1^ and (4.8 ± 1.0) × 10^5^ M^–1^ (Fig. S12 and S13†), respectively. In the case of phosphate, only the salt bridge interaction is presented; in the case of 6 : 0 LPA, the hydrophobic interaction between alkyl chains of GC5A and LPA is lacking. Thus, they were merely able to reach mM to μM binding.

Moreover, two control hosts (GC4A and GC5A–CH_3_, see Scheme S3† for their syntheses and structures) were prepared to illustrate the significance of cavity size and rigid cone conformation. The C4A cavity is too small to thread any alkyl chains.13*a* GC4A affords over one order of magnitude weaker affinity ((1.3 ± 0.1) × 10^5^ M^–1^) to Fl than GC5A (Fig. S14 and S15†). By executing IDA, gradual addition of LPA does not lead to pronounced regeneration of fluorescence in the beginning, indicating the weak complexation of GC4A with LPA (Fig. S16†). However, in the presence of excess LPA, fluorescence was regenerated. We postulated that it is not the *endo*-complexation but the co-assembly between cationic (GC4A) and anionic (LPA) surfactants that leads to weak complexation.^18^ The formation of the co-assembly between GC4A and LPA was verified by dynamic light scattering (DLS) measurements (Fig. S17†). The scattering intensity gradually increases upon addition of excess LPA, which is in good accordance with the fluorescence result. It is worth noting that no appreciable DLS response was detected for all the direct and competitive titrations of GC5A. Benefiting from strong host–guest complexation, the titrations were performed at sub-μM concentrations and no co-assembly was formed. GC5A–CH_3_, the control C5A host with conformational flexibility, quenches the fluorescence of Fl to a much lower extent and affords the corresponding weaker affinity ((4.5 ± 0.3) × 10^4^ M^–1^) than GC5A (Fig. S18†). Therefore, both the cavity size and conformational rigidification play crucial roles in molecular recognition. The employment of C5A and the well-tailored modification are indispensable in realizing nanomolar binding of GC5A with LPA.

**Fig. 2:**
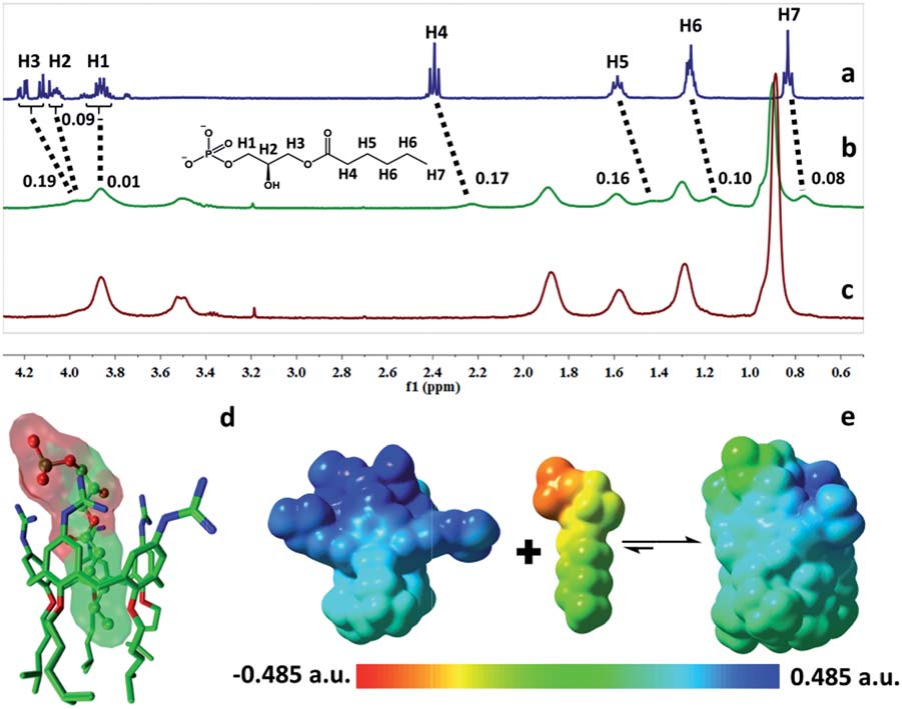
^1^H NMR spectra of (a) 6:0 LPA (1 mM), (b) 6:0 LPA (1 mM) with addition of GC5A (1 mM), and (c) GC5A (1 mM) in D_2_O at 25 °C. (d) Optimized structure of the GC5A·6:0 LPA complex at the B3LYP-D3(BJ)/6-31G(d)/SMD(water) level of theory. Hydrogen atoms are omitted for clarity. (e) ESP-mapped molecular vdW surface of GC5A, 6:0 LPA and GC5A·6:0 LPA.

The complexation of LPA with GC5A was further verified by ^1^H NMR experiments in D_2_O. We employed the shorter 6 : 0 LPA as the model guest for NMR measurements due to the poor water-solubility of the longer LPA species. LPA protons underwent upfield shifts upon addition of GC5A (Fig. 2a–c) due to the ring current effect of the aromatic nuclei of calixarenes.^13^,^19^ It should be mentioned that the complexation-induced shifts of guest protons by GC5A are much less pronounced than those in the other calixarene cases (generally Δ*δ* = 1–2 ppm).11c,13a This is due to the low molecular electrostatic potential of GC5A (*vide infra*), leading to a relatively weak ring current effect. The shifts of H3–H5 signals are larger than the rest, indicating their location in the center of the cavity. However, H1 and H2 are presumably located at the upper rim, H6 and H7 are close to the lower-rim oxygen mean plane and thread out of the cavity, which are away from the region of maximum shielding provided by the aromatic rings. Moreover, NMR measurements were performed above CAC, so it is also possible that GC5A and LPA form a co-assembly, but not an *endo*-complex. If GC5A forms a co-assembly with LPA, it is possible that GC4A also forms a co-assembly. We further executed the NMR measurements of GC4A with LPA as a control, observing no appreciable complexation-induced shifts (Fig. S20†). We therefore deduced that the shifts of LPA protons arose from the *endo*-complexation by GC5A.

Geometry optimization on the GC5A·6:0 LPA complex was performed using the B3LYP-D3(BJ)/6-31G(d)/SMD(water) method.^20^ The complex has a threading geometry (Fig. 2d), which is in good accord with the NMR information. To derive further insights for the host–guest binding, we have computed molecular electrostatic potential^21^ (ESP) mapped on the molecular van der Waals (vdW) surface of GC5A, 6:0 LPA and the GC5A·6:0 LPA complex (Fig. 2e). GC5A is highly electron-deficient particularly at the upper rim, while LPA is electron-rich, particularly at the phosphate head. The binding mode between GC5A and LPA is favorable because molecules always tend to approach each other in a complementary manner of ESP. Furthermore, the expected hydrogen bonds, C–H···π, C–H···O and vdW interactions between GC5A and LPA were validated by atoms-in-molecules and reduced density gradients analysis (see ESI†). Geometry optimization on the GC5A·18:0 LPA complex provides reasonably consistent results (Fig. S30†).

The IDA principle based on the GC5A·Fl reporter pair allows for a fluorescence “switch-on” sensing of LPA. As shown in Fig. S21,† the fluorescence increases linearly with LPA concentration with good linear performance (*R*^2^ = 0.997). The limit of detection (LOD) for LPA is calculated to be 5.6 nM by utilizing a 3*σ*/slope method,^22^ which is far lower than the requisite detection limit in plasma.^3^ Compared with the currently reported fluorescent probes,6a–c 5.6 nM represents the lowest LOD value in LPA detection, indicating the ultrahigh sensitivity of GC5A·Fl.

**Fig. 3:**
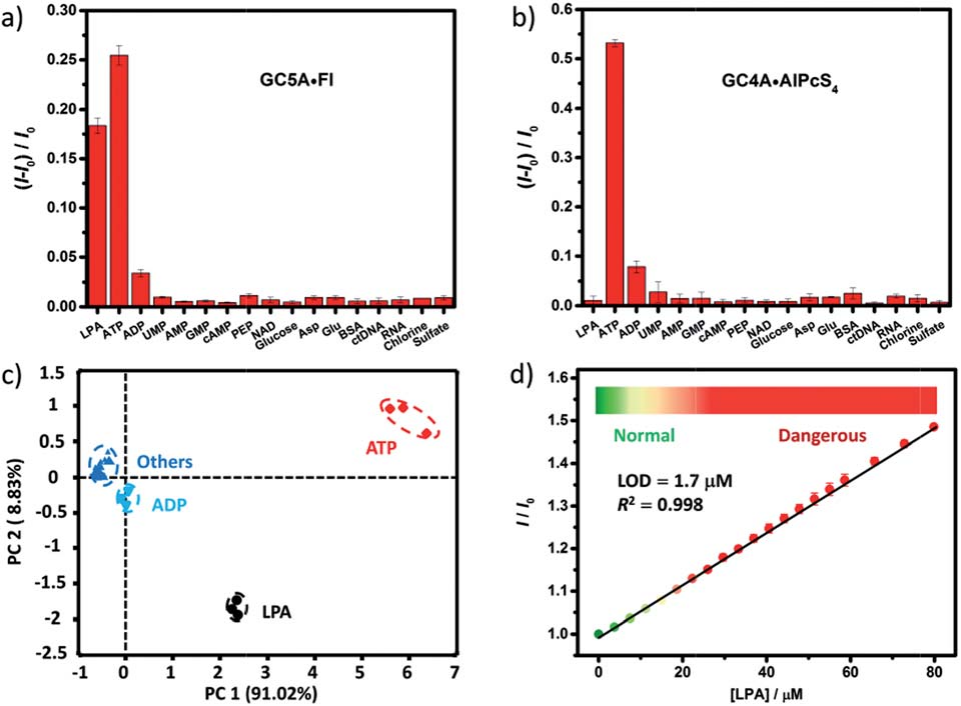
Fluorescence responses of (a) GC5A·Fl (0.8/1.0 μM) at 513 nm (*λ*_ex_ = 500 nm) and (b) GC4A·AlPcS_4_ (0.8/1.0 μM) at 680 nm (*λ*_ex_ = 608 nm) upon the addition of LPA and various biological co-existing species (0.4 μM for small species and 0.15 mg L^–1^ for ctDNA, RNA and BSA) in HEPES buffer. (c) Score plot of the first two principal components obtained by PCA of analytes. The percent of total variance is given in brackets for each principal component. Ellipsoids on the scatter plot are drawn at 95% confidence. (d) The set-up calibration line of the fluorescence intensity for quantitatively determining the LPA concentrations in serum. Error bars could not be shown if less than 0.005.

We further tested changes in the fluorescence intensity of GC5A·Fl caused by other biologically important species (nucleoside polyphosphates, amino acids, anions, carbohydrate, ctDNA, RNA and BSA) in plasma to evaluate the sensing selectivity for LPA (Fig. 3a). In most cases, the addition of other biological species caused no significant increase in the fluorescence. The only exception was ATP, which resulted in even more pronounced fluorescence response than LPA due to the strong binding of ATP with GC5A (*K*_a_ = (4.7 ± 1.4) × 10^8^ M^–1^, Fig. S24†). Such an interference could be easily solved by differential sensing. Differential sensing relies on the composite response of the analyte to the entire array of receptors instead of a single receptor; hence, it is also called “array sensing”, providing output with better accuracy and more robust interference resistance. Although both the direct sensing and IDA approaches can be used, IDA is more compatible with differential sensing because an array can be easily constructed by the combination of multiple receptors and multiple indicators without additional synthetic efforts.17*b* Herein, GC4A was additionally introduced as a receptor and Al(iii) phthalocyanine chloride tetrasulfonic acid (AlPcS_4_, Scheme S4†) was introduced as an additional reporter dye. AlPcS_4_ is also strongly bound by GC5A and GC4A with drastic fluorescence quenching (Fig. S22†). We therefore used GC5A·Fl, GC5A·AlPcS_4_ and GC4A·AlPcS_4_ as reporter pairs for differential sensing to differentiate LPA from other species in plasma through the different fluorescence response pattern (Fig. 3a, b and S25†). Executing principal component analysis (PCA), a statistical method to find the greatest extent of variance in a dataset, resulted in a score plot (Fig. 3c).^23^ LPA was definitely distinguished from ATP and the other coexisting species. The major rationale behind the present differential sensing is that the threading complex of LPA could be only formed by GC5A and not by GC4A. The ratio of fluorescence response (*I*_LPA_/*I*_ATP_) in the case of GC5A is thus much larger than that in the case of GC4A.

To validate the practical operational detection of LPA, we performed the displacement assay of LPA in mouse serum containing variable LPA concentrations. Despite the existence of numerous interfering substances in the serum, a linear increase in the fluorescence of the GC5A·AlPcS_4_ reporter pair was still observed upon the gradual increase in LPA concentrations (0–80 μM) (Fig. 3d). The LOD in serum was calculated as 1.7 μM, which is well below plasma LPA concentrations typically observed in patients with ovarian and other gynecologic cancers. The good linear relationship (*R*^2^ = 0.998) allows us to construct a calibration line for the fluorescence intensity to accurately determine unknown concentrations of LPA down to the low μM range, which is of practical diagnostic relevance. Furthermore, we applied the GC5A·AlPcS_4_ reporter pair in analyzing cancerous and non-cancerous blood samples. The blood samples were obtained from healthy mice and mice with ovarian tumour, which was created by inoculating ID8 cells subcutaneously. A significant difference was observed, whereby the cancerous group had a greater fluorescence response than the non-cancerous group (Fig. S26†). This result validates that the present IDA protocol has great potential in facilitating the practical operational diagnosis of ovarian cancer.

## Conclusions

In conclusion, we designed an artificial receptor GC5A for LPA, a cancer biomarker, with nanomolar affinity in aqueous media. Through IDA coupled with differential sensing, we achieved ultrasensitive and specific detection of LPA. For accurately determining unknown concentrations of LPA down to the low μM range, which is of practical diagnostic relevance, a calibration line was successfully set up in serum. To the best of our knowledge, although calibration lines of LPA have been obtained among several known assay approaches,4a,6f,g,^24^ the present approach represents the first example obtained in untreated serum. These results form the chemical basis for new protocols and devices to diagnose ovarian cancer and other gynecologic cancers, especially during their early stages.

All animal studies were performed in compliance with the guidelines set by the Tianjin Committee of Use and Care of Laboratory Animals and the overall project protocols were approved by the Animal Ethics Committee of Nankai University.

## Conflicts of interest

There are no conflicts to declare.

## Supplementary Material

Supplementary information
