# Supplementary material for: Ultrasensitive and specific fluorescence detection of a cancer biomarker via nanomolar binding to a guanidinium-modified calixarene
Source: Chem Sci. 2018 Jan 9;9(8):2087–91. doi: 10.1039/c7sc04989g (PMC5892409; doi:10.1039/c7sc04989g)
Supplement: Supplementary file 1 [file SC-009-C7SC04989G-s001.pdf]

Electronic Supplementary Material (ESI) for Chemical Science.
This journal is © The Royal Society of Chemistry 2018

Electronic Supplementary Information

for

Ultrasensitive and Specific Fluorescence Detection of a Cancer Biomarker via Nanomolar Binding to a Guanidinium-modified Calixarene

Zhe Zheng,[a] Wen-Chao Geng,[a] Jie Gao,[a] Yu-Ying Wang,[a] Hongwei Sun[a] and Dong-Sheng Guo*[a,b]

[a] *College of Chemistry, State Key Laboratory of Elemento-Organic Chemistry, Key Laboratory of Functional Polymer Materials, Ministry of Education, Nankai University, Tianjin 300071, China. Email: dshguo@nankai.edu.cn*

[b] *Collaborative Innovation Center of Chemical Science and Engineering, Nankai University, Tianjin 300071, China.*

Table of Contents

1. **General methods and materials** 1

2. **Synthesis of GC5A and GC5A-CH_3** 2

3. **Supporting results and experimental raw data** 12

3.1 Structures of LPAs, two control hosts and $AlPcS_4$. 12
3.2 The critical aggregation concentration (CAC) of GC5A determined by DLS 13
3.3 Job's plot analysis for the complexation of Fl with GC5A 13
3.4 UV-Vis titration of Fl with GC5A 14
3.5 Competitive fluorescence titration of phosphate and 6:0 LPA with GC5A 14
3.6 Fluorescence, UV-Vis and DLS measurements of Fl with GC4A 15
3.7 Fluorescence measurement of Fl with GC5A-CH_3 16
3.8 HMBC spectrum of 6:0 LPA 17
3.9 ^{1}H NMR spectra of GC4A and 6:0 LPA 17
3.10 The limit of detection (LOD) for LPA in HEPES buffer 18
3.11 Fluorescence measurements of $AlPcS_4$ with GC5A and GC4A 18
3.12 Fluorescence responses of various biologically coexisting species with GC5A and GC4A 19
3.13 Analyzing cancerous and non-cancerous blood samples of mice by the GC5A•$AlPcS_4$ reporter pair 20

4. **Theoretical Calculations** 20

5. **Reference** 25

1. General methods and materials

All the reagents and solvents were commercially available and used as received unless otherwise specified purification. Lysophosphatidic acids (sodium salt) (LPAs) were purchased from Avanti. Poly A ribonucleic acid (RNA), calf thymus deoxyribonucleic acid (ctDNA), adenosine triphosphate (ATP), adenosine diphosphate (ADP), cyclic adenosine monophosphate (cAMP), adenosine monophosphate (AMP), uridine monophosphate (UMP) and guanosine monophosphate (GMP) were purchased from Sigma. Bovine serum albumin (BSA) and phosphoenolpyruvate (PEP) were purchased from J&K Chemical. Fluorescein (Fl), glutamic acid (Glu) and aspartic acid (Asp) were purchased from Tokyo Chemical Industry. Nicotinamide adenine dinucleotide (NAD) and glucose were purchased from Aladdin. Al(III) phthalocyanine chloride tetrasulfonic acid ($AlPcS_4$) was purchased from Frontier Scientific. 5,11,17,23-Tetraguanidinium-25,26,27,28-tetrabutoxycalix[4]arene (GC4A) was synthesized according to the previous literature.[1]

The HEPES buffer solution of pH 7.4 was prepared by dissolving 2.38 g of 2-[4-(2-hydroxyethyl)piperazin-1-yl]ethanesulfonic acid (HEPES) in approximate 900 mL double-distilled water. Titrate to pH 7.4 at the lab temperature of 25 °C with NaOH and make up volume to 1000 mL with double-distilled water. The pH value of the buffer solution was then verified on a pH-meter calibrated with three standard buffer solutions. All fluorescence and UV-Vis titrations were measured in HEPES buffer (10 mM, pH 7.4) at 25 °C.

The mouse serum was purchased from Manuik. The set-up calibration line of the fluorescence intensity for quantitatively determining the LPA concentrations was carried out in tenfold diluted mouse serum solutions with HEPES buffer (10 mM, pH 7.4) containing 5.0 μM $AlPcS_4$ and 10.0 μM GC5A.

For tumour model, all animal studies were performed in compliance with the guidelines set by Tianjin Committee of Use and Care of Laboratory Animals and the overall project protocols were approved by the Animal Ethics Committee of Nankai University. Female C57BL/6J healthy and tumour mice (6 weeks old) were purchased from Anti-hela Biological TechnologyTrade Co.Ltd (Xiamen, China). Female C57BL/6J tumour mice were implanted subcutaneously with ID8 cells.

All mean values from fluorescence and UV-Vis titrations, limit of detection and calibration lines were measured from at least three experiments and errors were given as standard deviation ($\pm 1\sigma$).

^1H, ^{13}C and ^1H-detected multiple-bond correlation (HMBC) NMR data were recorded on a Bruker AV400 spectrometer. High resolution mass spectra (HRMS) were performed on a VG ZAB-HS (ESI). UV-Vis spectra were recorded in a quartz cell (light path 10 mm) on a Cary 100 UV-Vis spectrophotometer equipped with a Cary dual cell peltier accessory. Steady-state fluorescence spectra were recorded in a conventional quartz cell (light path 10 mm) on a Cary Eclipse equipped with a Cary single-cell peltier accessory. The sample solutions for dynamic light scattering (DLS) measurements were examined on a laser light scattering spectrometer (NanoBrook

173plus) equipped with a digital correlator at 659 nm at a scattering angle of 90°.

2. Synthesis of GC5A and GC5A-CH$_3$

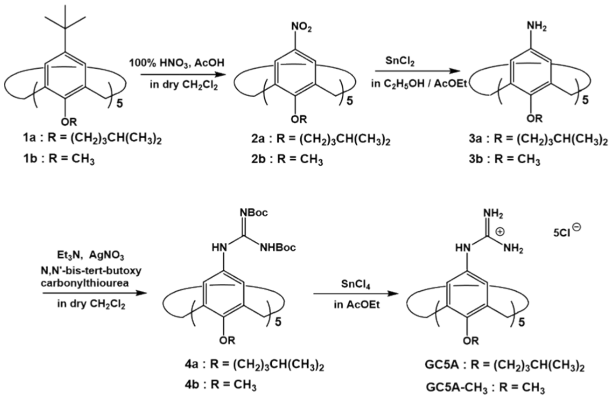

Scheme S1. Synthetic routes of **GC5A** and **GC5A-CH$_3$**.

5,11,17,23,29-Penta-tert-butyl-31,32,33,34,35-penta(4-methylpentyloxy)calix[5]arene (1a) was synthesized and purified according to procedures reported previously.[2]

Synthesis of 5,11,17,23,29-pentanitro-31,32,33,34,35-penta(4-methylpentloxy) calix[5]arene (2a):

To a solution of **1a** (3.96 g, 2.4 mmol) in dry CH_2Cl_2 (119 mL) and AcOH (34.28 mL), HNO_3 (10.28 mL) was added gradually and the mixture was stirred for about 1–4 h at room temperature. The color of the mixture changed from dark purple to orange. Then water (250 mL) was added into the reaction mixture, followed by stirring for 30 min. The mixture was washed with saturated Na_2CO_3, brine and water. After drying with Na_2SO_4, the solvent was removed in vacuo. The residues were recrystallized from CH_2Cl_2/CH_3OH to obtain pale yellow solid **2a** (1.74 g, 46%). ^{1}H NMR (400 MHz, $CDCl_3$, δ): 7.83 (s, 10H, ArH), 4.59 (d, J = 14.78 Hz, 5H, Ar-CH$_2$-Ar), 3.84 (t, J = 7.60 Hz, 10H, CH$_2$-O-Ar), 3.56 (d, J = 14.81 Hz, 5H, Ar-CH$_2$-Ar), 1.84 (m, 10H, -CH_2-CH$_2$-CH-), 1.60 (m, 5H, -CH-), 1.26 (m, 10H, -CH$_2$-CH_2-CH-), 0.95 (d, J = 6.59 Hz, 30H, -(CH$_3$)$_2$).

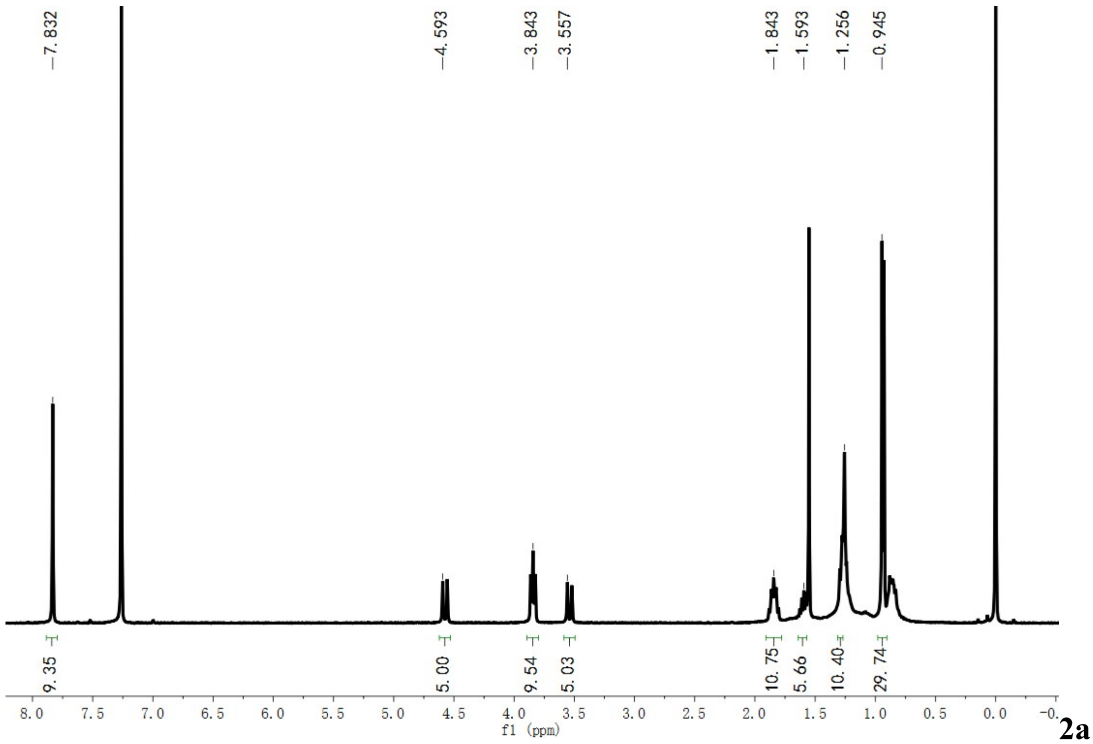

2a

Figure S1. ^{1}H NMR spectrum of **2a** in $CDCl_3$, 400 MHz, 25 °C.

Synthesis of 5,11,17,23,29-pentaamino-31,32,33,34,35-penta(4-methylpentloxy) calix[5]arene (3a):

To a solution of **2a** (0.28 g, 0.24 mmol) in dry C_2H_5OH/AcOEt (50 mL/50 mL), $SnCl_2{\cdot}2H_2O$ (1.5 g, 6.7 mmol) was added and the mixture was refluxed for 48 h. Then the mixture was pour into ice water. After all the ice melt, NaOH was added to the mixture to adjust the pH to 8.0. Dichloromethane was added and the mixture was stirred overnight at room temperature. The solution was washed by water for three times. After drying with Na_2SO_4, the solvent was removed in vacuo to obtain white solid **3a** (0.13 g, 52%). ^{1}H NMR (400 MHz, $CDCl_3$, δ): 6.17 (s, 10H, ArH), 4.40 (d, J = 14.42 Hz, 5H, Ar-CH_2-Ar), 3.60 (t, J = 7.28 Hz, 10H, CH_2-O-Ar), 3.11 (d, J = 14.25 Hz, 5H, Ar-CH_2-Ar), 1.74 (q, J = 7.57 Hz, 10H, -CH_2-CH_2-CH-), 1.56 (m, 5H, -CH-), 1.27 (m, 10H, -CH_2-CH_2-CH-), 0.91 (d, J = 6.56 Hz, 30H, -$(CH_3)_2$).

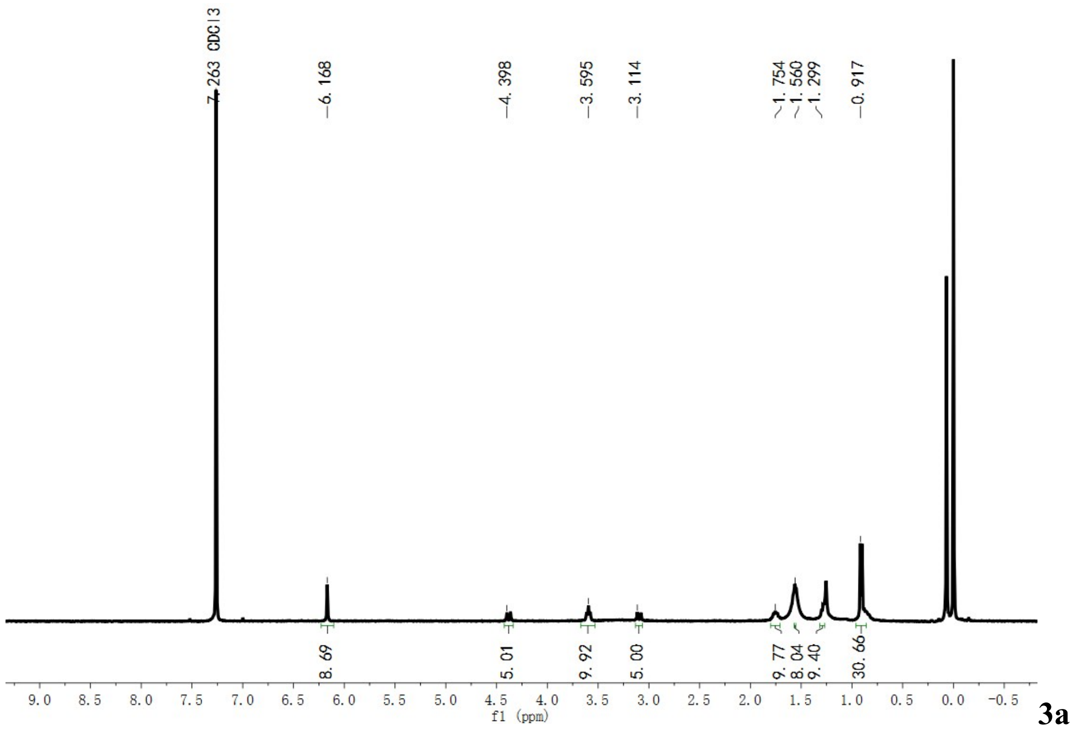

3a

Figure S2. ^{1}H NMR spectrum of **3a** in $CDCl_3$, 400 MHz, 25 °C.

Synthesis of 5,11,17,23,29-penta[(bis-N-Boc)guanidine]-31,32,33,34,35-penta (4-methylpentloxy)calix[5]arene (4a):

To a solution of **3a** (0.20 g, 0.22 mmol) in dry CH_2Cl_2 (25 mL), N,N'-bis-tert-butoxycarbonylthiourea (0.33 g, 1.12 mmol), $AgNO_3$ (0.20 g, 1.19 mmol) and Et_3N (0.17 mL) were added and the mixture was stirred for 48 h at room temperature. The solvent was removed in vacuo and the residue was purified by column chromatography on silica gel to obtain a pale white powder **4a** (0.17 g, 32%). ^{1}H NMR (400 MHz, $CDCl_3$, δ): 11.63 (s, 5H, NH), 9.87 (s, 5H, NH), 7.14 (s, 10H, ArH), 4.54 (d, J = 13.58 Hz, 5H, Ar-CH_2-Ar), 3.75 (t, J = 7.76 Hz, 10H, CH_2-O-Ar), 3.30 (d, J = 13.81 Hz, 5H, Ar-CH_2-Ar), 1.91 (m, 10H, -CH_2-CH_2-CH-), 1.60 (m, 5H, -CH-), 1.46 (d, 90H, J = 13.81 Hz , Bu^t), 1.15 (m, 10H, -CH_2-CH_2-CH-), 0.95 (d, J = 6.58 Hz, 30H, -$(CH_3)_2$).

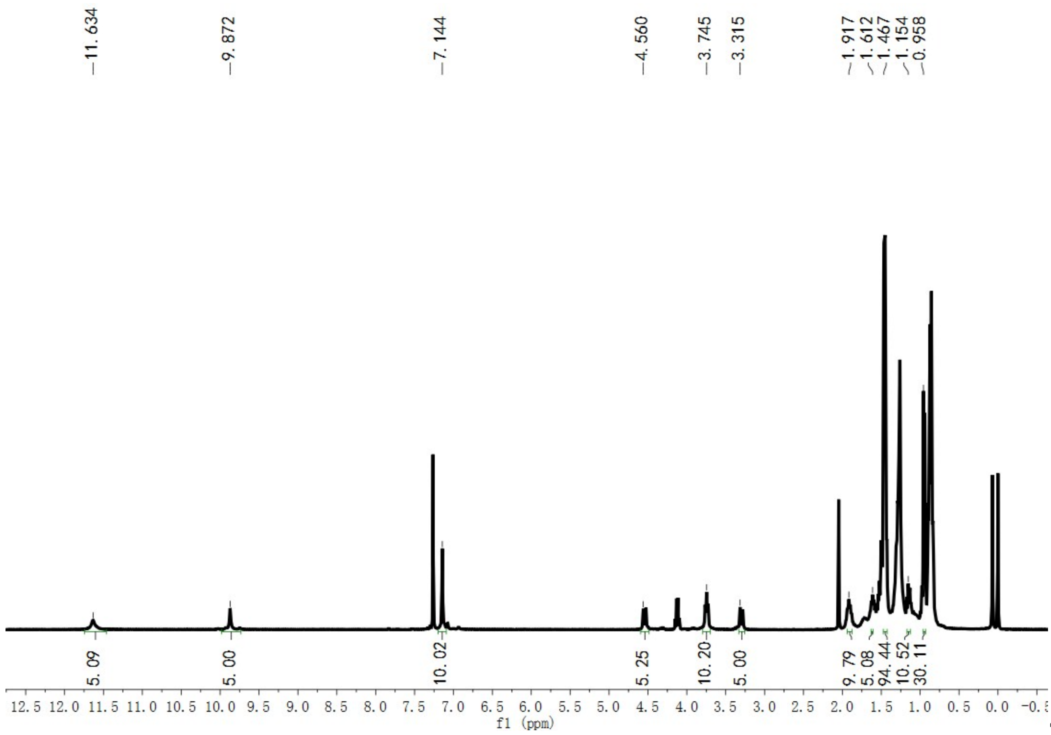

4a

Figure S3. ^{1}H NMR spectrum of **4a** in $CDCl_3$, 400 MHz, 25 °C.

Synthesis of 5,11,17,23,29-pentaguanidinium-31,32,33,34,35-penta (4-methylpentloxy)calix[5]arene (GC5A):

$SnCl_4$ (0.2 mL) was added to a solution of **4a** (0.08 g, 0.03 mmol) in 20 mL of AcOEt. The mixture was stirred for 3 h at room temperature and the solvent was removed in vacuo. The residue was dissolved in CH_3OH, then large amounts of diethyl ether was added to obtain white powder **GC5A** (0.03 g, 65%). ^{1}H NMR (400 MHz, DMSO, δ): 9.62 (s, 5H, NH), 7.35 (s, 20H, NH), 6.95 (s, 10H, ArH), 4.46 (d, J = 13.61 Hz, 5H, Ar-CH_2-Ar), 3.80 (t, J = 7.74 Hz, 10H, CH_2-O-Ar), 3.46 (d, J = 13.58 Hz, 5H; Ar-CH_2-Ar), 1.91 (p, J = 7.54 Hz, 10H, -CH_2-CH_2-CH-), 1.61 (m, 5H, -CH-), 1.32 (m, 10H, -CH_2-CH_2-CH-), 0.94 (d, J = 6.61 Hz, 30H, -$(CH_3)_2$). ^{13}C NMR (100 MHz, DMSO, δ): 155.82, 153.41, 134.62, 129.67, 125.57, 74.22, 64.54, 34.64, 27.65, 22.56, 15.19. ESI-FTMS m/z: $[M + H - 5HCl]^+$ calcd. for $C_{70}H_{106}N_{15}O_5^+$ 1236.8496, found 1236.8472.

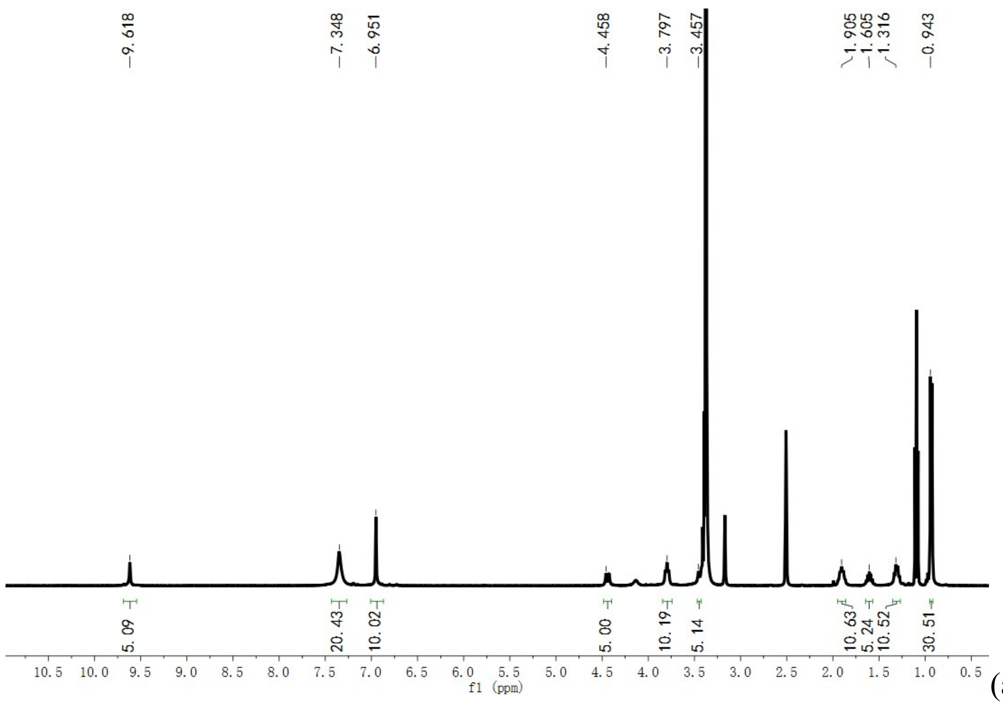

9.618
7.348
6.951
4.458
3.797
3.457
1.905
1.605
1.316
0.943
5.09
20.43
10.02
5.00
10.19
5.14
10.63
5.24
10.52
30.51
f1 (ppm)

(a)

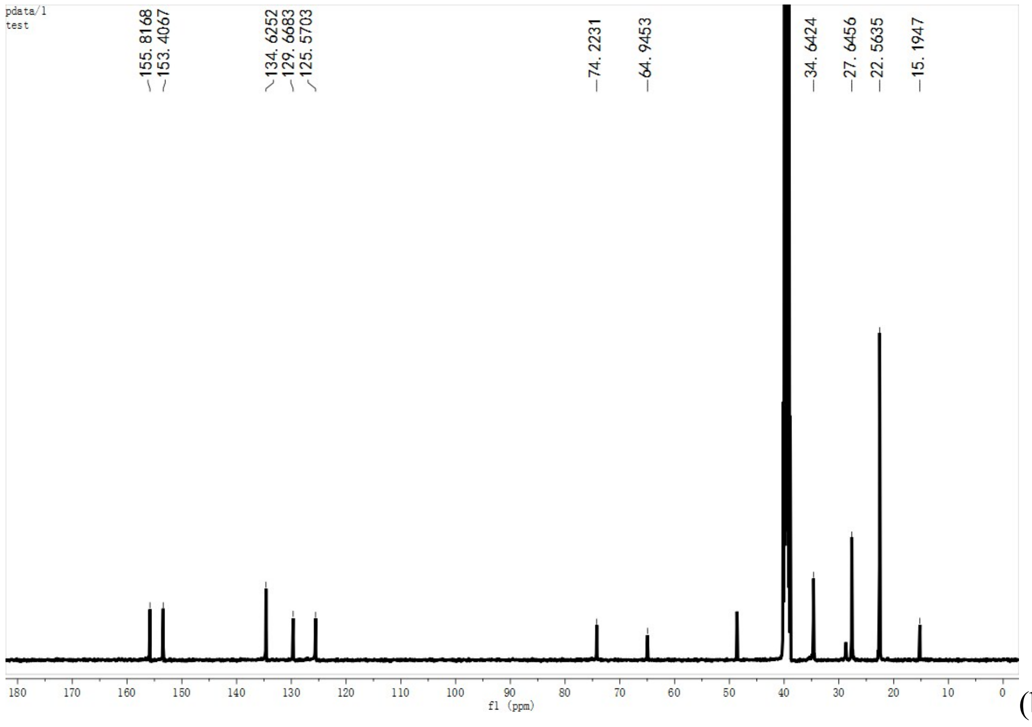

pdata/1
test
155.8168
153.4067
134.6252
129.6683
125.5703
74.2231
64.9453
34.6424
27.6456
22.5635
15.1947
f1 (ppm)

(b)

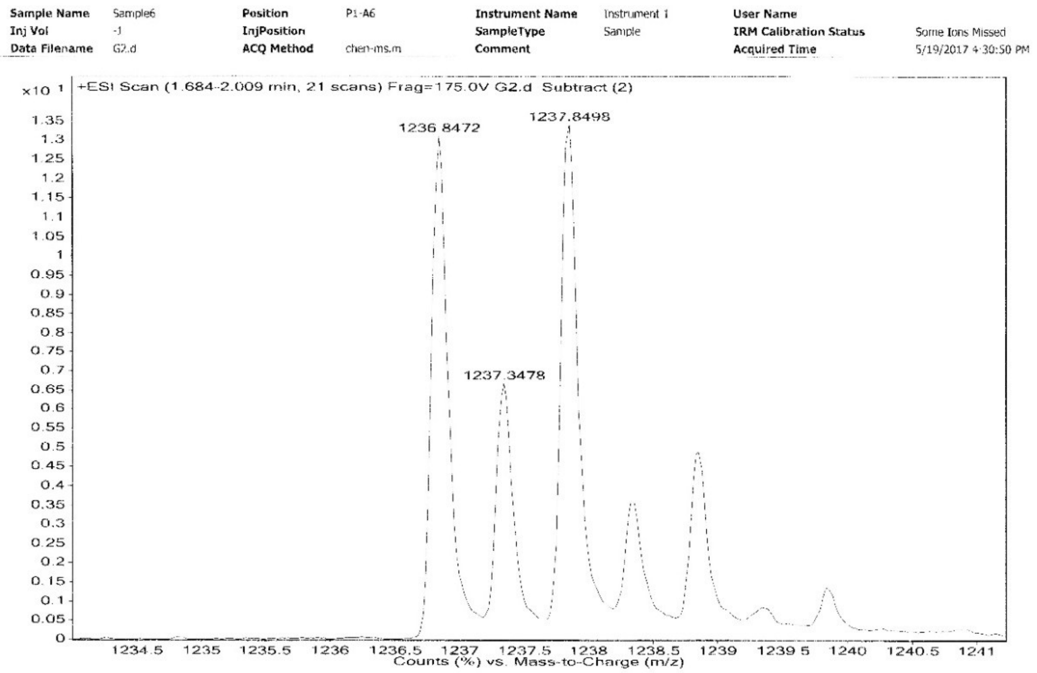

(c)

Figure S4. (a) ^{1}H NMR spectrum of **GC5A** in DMSO, 400 MHz, 25 °C; (b) ^{13}C NMR spectrum of **GC5A** in DMSO, 100 MHz, 25 °C; (c) electrospray ionization fourier transform ion cyclotron resonance-MS of **GC5A**.

5,11,17,23,29-Penta-tert-butyl-31,32,33,34,35-pentamethoxycalix[5]arene (1b) were synthesized and purified according to procedures reported previously.[3]

Synthesis of 5,11,17,23,29-pentanitro-31,32,33,34,35-pentamethoxycalix[5]arene (2b):

To a solution of **1b** (0.2 g, 0.2 mmol) in dry CH_2Cl_2 (20 mL) and AcOH (2.86 mL), HNO_3 (0.9 mL) was added gradually and the mixture was stirred for about 1~4 h at room temperature. The color of the mixture changed from dark purple to orange. Then water (18 mL) was added into the reaction mixture and continuously stirred for 30 min. The mixture was washed with saturated Na_2CO_3, brine and water. After drying with Na_2SO_4, the solvent was removed in vacuo. The residues were recrystallized from CH_2Cl_2/CH_3OH to obtain pale yellow solid **2b** (0.09 g, 48%). ^{1}H NMR (400 MHz, $CDCl_3$, δ): 7.96 (s, 10H, ArH), 3.94 (s, 10H, Ar-CH_2-Ar), 3.38 (s, 15H, -O-CH_3).

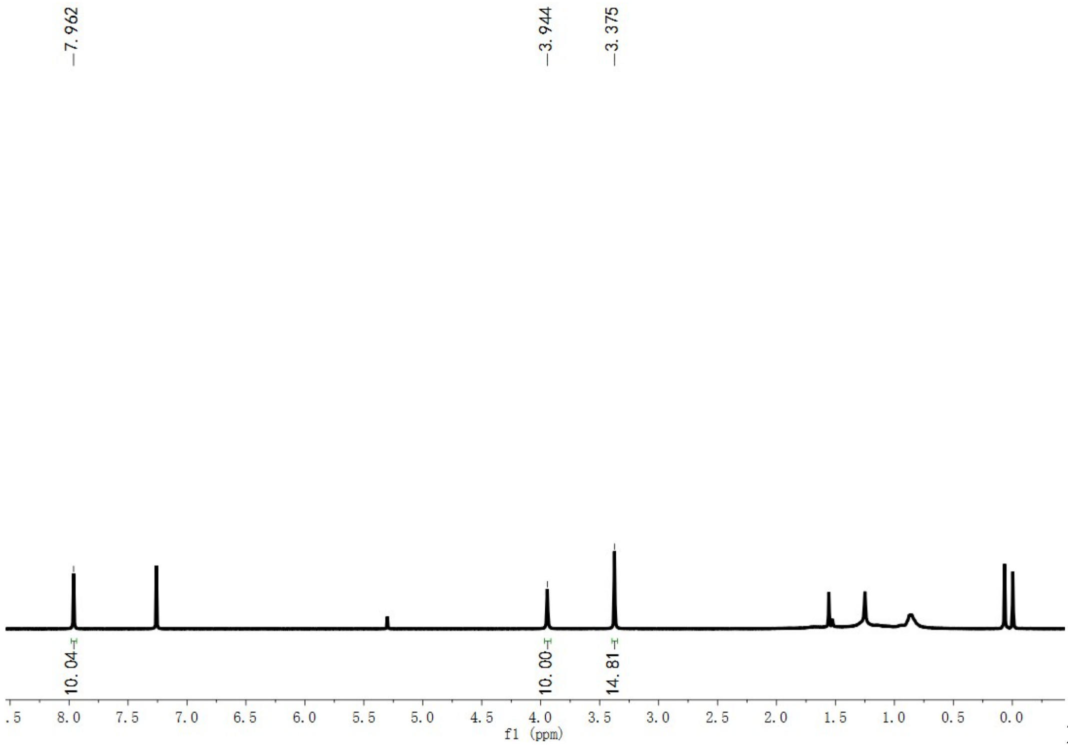

2b

Figure S5. ^{1}H NMR spectrum of **2b** in $CDCl_3$, 400 MHz, 25 °C.

Synthesis of 5,11,17,23,29-pentaamino-31,32,33,34,35-pentamethoxycalix[5]arene (3b):

To a solution of **2b** (0.1 g, 0.24 mmol) in dry C_2H_5OH/AcOEt (13 mL/13 mL), $SnCl_2{\cdot}2H_2O$ (1.5 g, 6.7 mmol) was added and the mixture was refluxed for 48 h. Then the mixture was pour into ice water. After all the ice melt, NaOH was added to the mixture to adjust the pH to 8.0. Dichloromethane was added and the mixture was stirred overnight at room temperature. The solution was washed by water for three times. After drying with Na_2SO_4, the solvent was removed in vacuo to obtain white solid **3b** (0.05 g, 56%). ^{1}H NMR (400 MHz, $CDCl_3$, δ): 6.30 (s, 10H, ArH), 3.71 (s, 10H, Ar-CH_2-Ar), 3.20 (s, 15H, -O-CH_3).

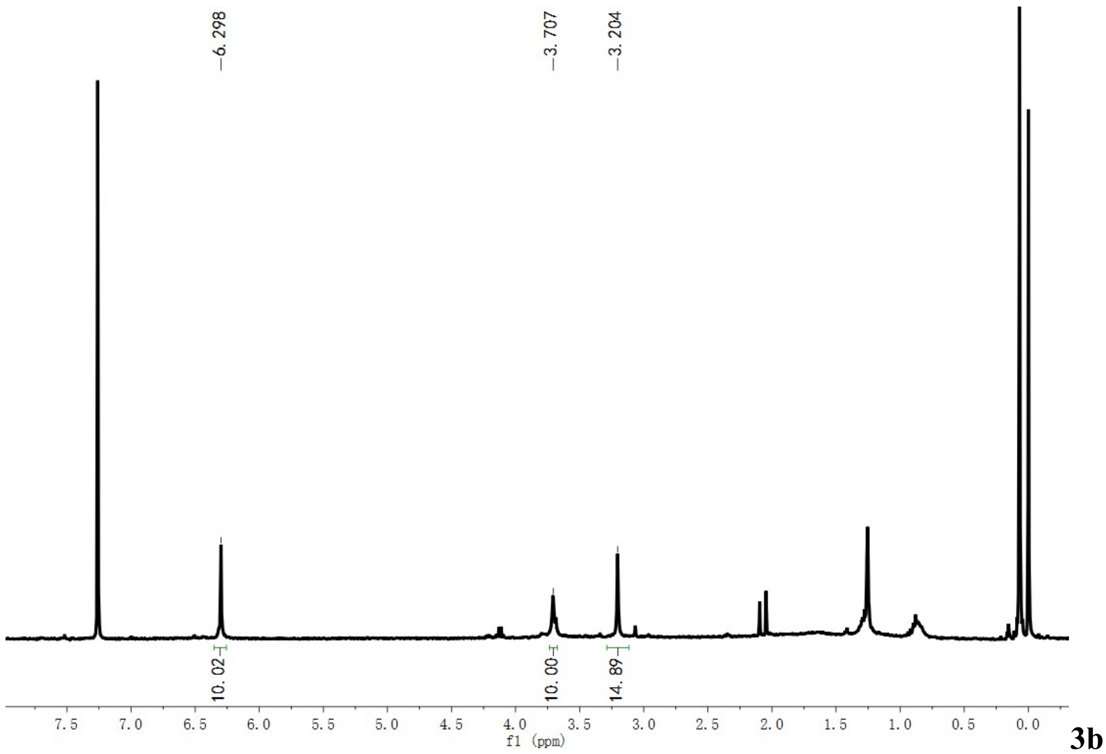

3b

Figure S6. ^1H NMR spectrum of **3b** in $CDCl_3$, 400 MHz, 25 °C.

Synthesis of 5,11,17,23,29-penta[(bis-N-Boc)guanidine]-31,32,33,34,35-pentamethoxycalix[5]arene (4b):

To a solution of **3b** (0.10 g, 0.15 mmol) in dry CH_2Cl_2 (25 mL), N,N'-bis-tert-butoxycarbonylthiourea (0.21 g, 0.74 mmol), $AgNO_3$ (0.13 g, 0.74 mmol) and Et_3N (0.2 mL) were added and the mixture was stirred for 48 h at room temperature. The solvent was removed in vacuo and the residue was purified by column chromatography on silica gel to obtain a pale white powder **4b** (0.09 g, 34%). ^1H NMR (400 MHz, $CDCl_3$, δ): 11.62 (s, 5H, NH), 10.00 (s, 5H, NH), 7.20 (s, 10H, ArH), 3.81 (s, 10H, Ar-CH_2-Ar), 3.26 (s, 15H, -O-CH_3), 1.49 (s, 90H, Bu^t).

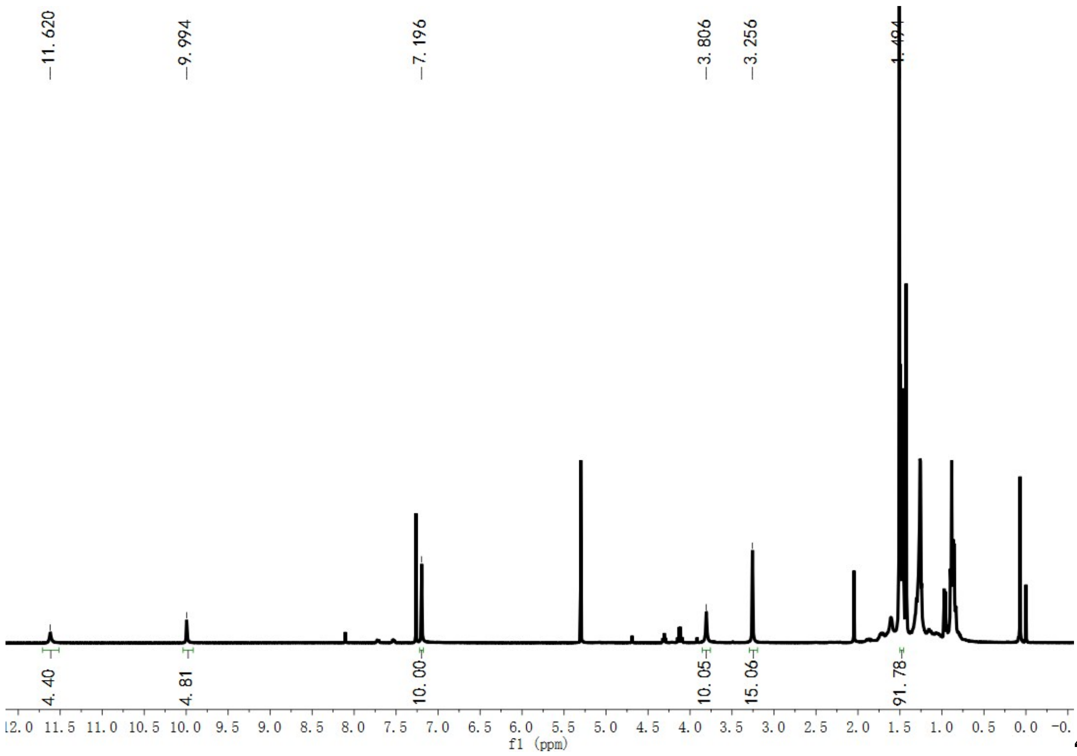

4b

Figure S7. ^{1}H NMR spectrum of **4b** in $CDCl_3$, 400 MHz, 25 °C.

Synthesis of 5,11,17,23,29-pentaguanidinium-31,32,33,34,35-pentamethoxycalix[5]arene (GC5A-CH_3):

$SnCl_4$ (0.2 mL) was added to a solution of **4b** (0.07 g, 0.03 mmol) in 20 mL of AcOEt. The mixture was stirred for 3 h at room temperature and the solvent was removed in vacuo. The residue was dissolved in CH_3OH, then large amounts of diethyl ether was added to obtain white powder **GC5A-CH_3** (0.03 g, 78%). ^{1}H NMR (400 MHz, DMSO, δ): 9.77 (s, 5H, NH), 7.33 (s, 20H, NH), 6.94 (s, 10H, ArH), 3.85 (s, 10H, Ar-CH_2-Ar), 3.32 (s, 15H, -O-CH_3). ^{13}C NMR (100 MHz, DMSO, δ): 156.30, 155.01, 135.46, 130.28, 125.93, 61.05, 30.12. ESI-FTMS *m/z*: [M + H – 5HCl] $^+$ calcd. for $C_{45}H_{55}N_{15}O_5^+$ 886.4575, found 886.4570.

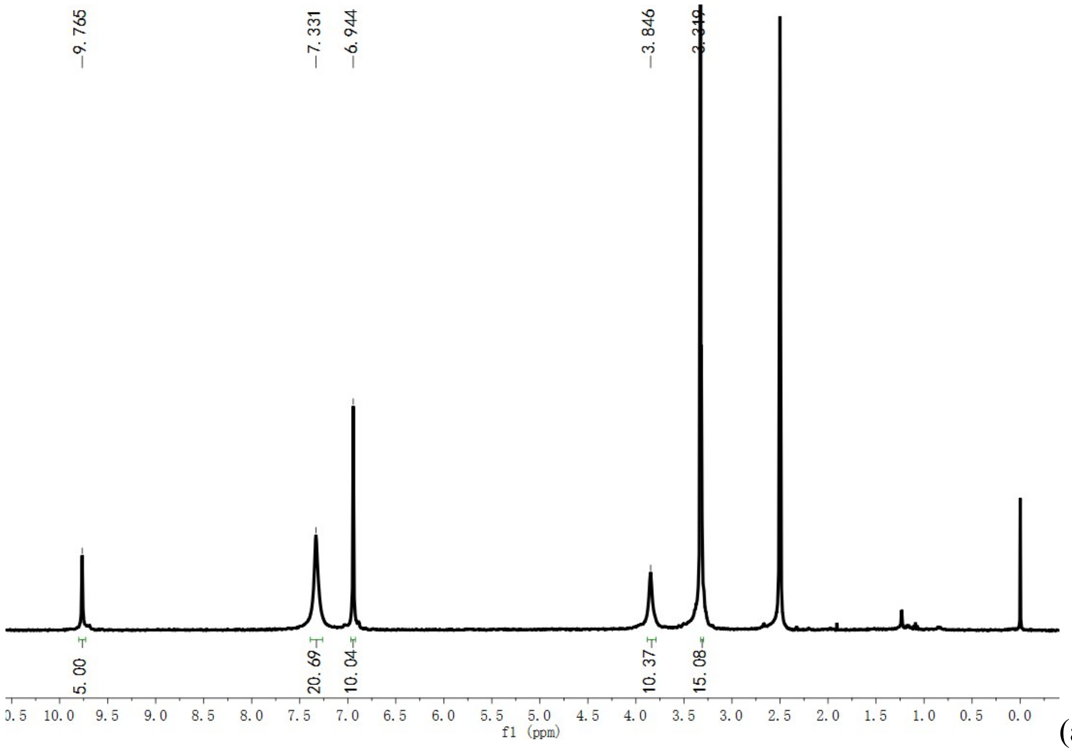
9.765
7.331
6.944
3.846
3.319
5.00
20.69
10.04
10.37
15.08
0.5 10.0 9.5 9.0 8.5 8.0 7.5 7.0 6.5 6.0 5.5 5.0 4.5 4.0 3.5 3.0 2.5 2.0 1.5 1.0 0.5 0.0
f1 (ppm)

(a)

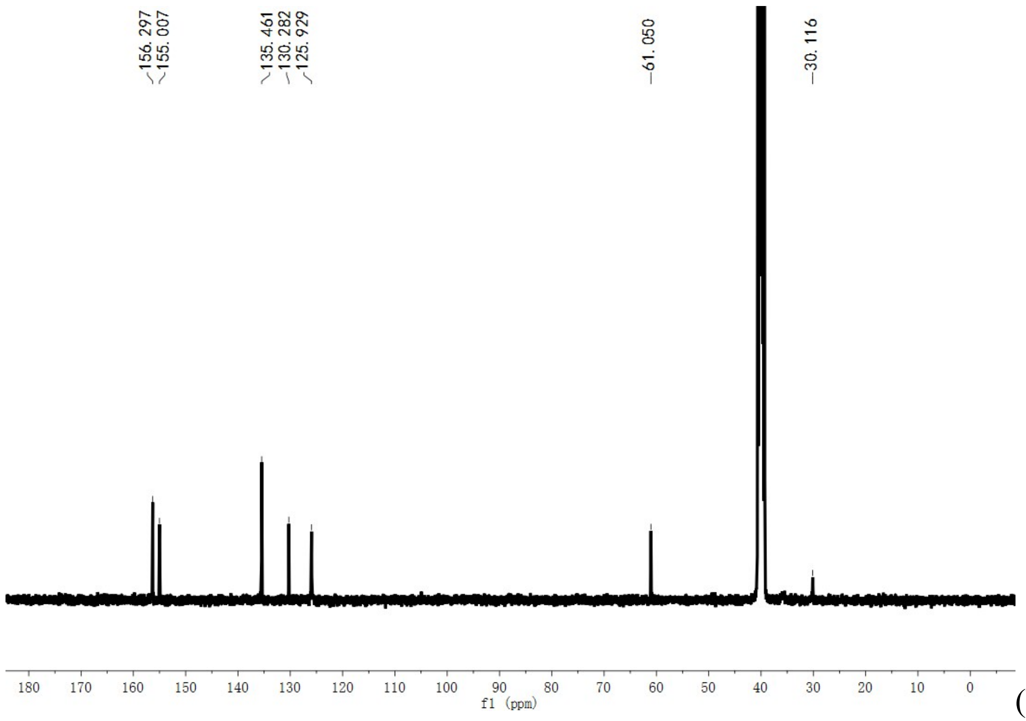
156.297
155.007
135.461
130.282
125.929
61.050
30.116
180 170 160 150 140 130 120 110 100 90 80 70 60 50 40 30 20 10 0
f1 (ppm)

(b)

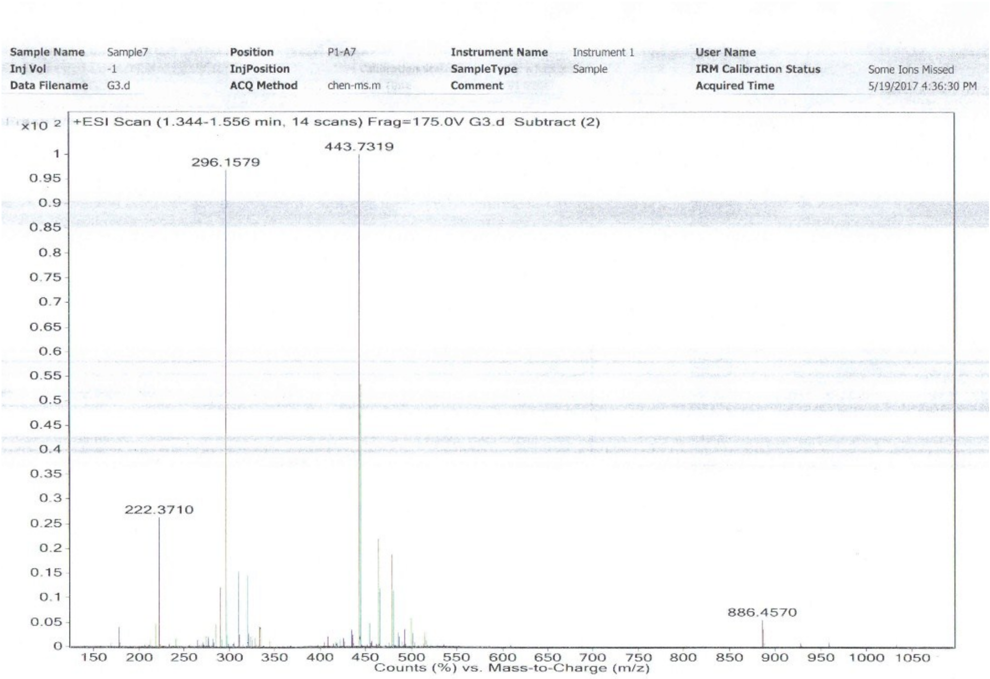

(c)

Figure S8. (a) ^{1}H NMR spectrum of **GC5A-CH$_3$** in DMSO, 400 MHz, 25 °C; (b) ^{13}C NMR spectrum of **GC5A-CH$_3$** in DMSO, 100 MHz, 25 °C; (c) Electrospray ionization fourier transform ion cyclotron resonance-MS of **GC5A-CH$_3$**.

3. Supporting results and experimental raw data

3.1 Structures of LPAs, two control hosts and AlPcS$_4$.

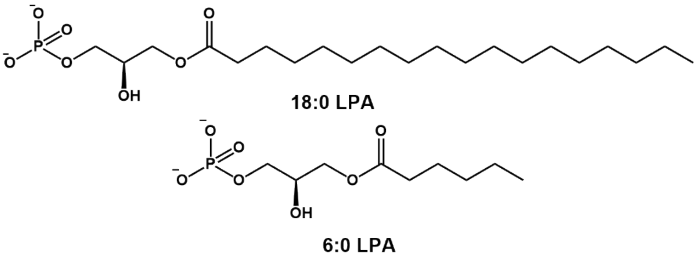

Scheme S2. Structures of 18:0 LPA and 6:0 LPA. 6:0 LPA was employed as a model guest to investigate the binding geometry by NMR measurements. All fluorescence titration measurements were performed by using 18:0 LPA (abbreviated as LPA in the main text for simplicity) as the typical analyte.

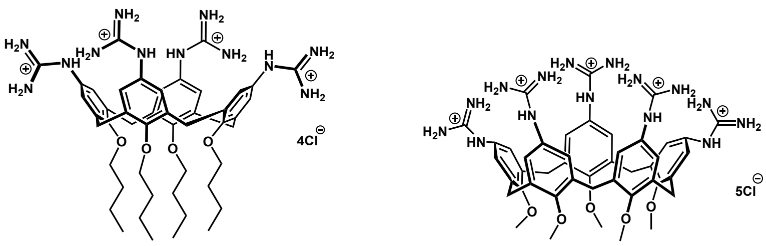

Scheme S3. Structures of GC4A and GC5A-CH$_3$.

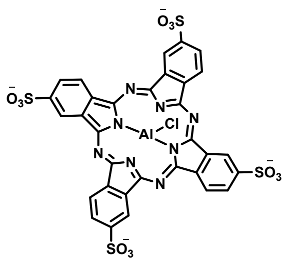

Scheme S4. Structure of $AlPcS_4$.

3.2 The critical aggregation concentration (CAC) of GC5A determined by DLS

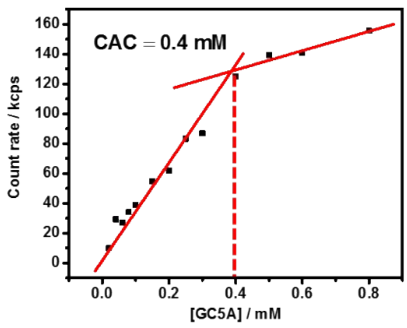

Figure S9. Dependence of light scattering intensity on the GC5A concentration in water at 25°C.

3.3 Job's plot analysis for the complexation of Fl with GC5A

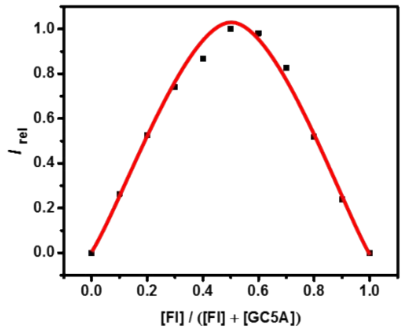

Figure S10. Job's plot for solutions of Fl and GC5A. λ_{ex} = 500 nm, λ_{em} = 513 nm, [Fl] + [GC5A] = 2.0 μM.

3.4 UV-Vis titration of Fl with GC5A

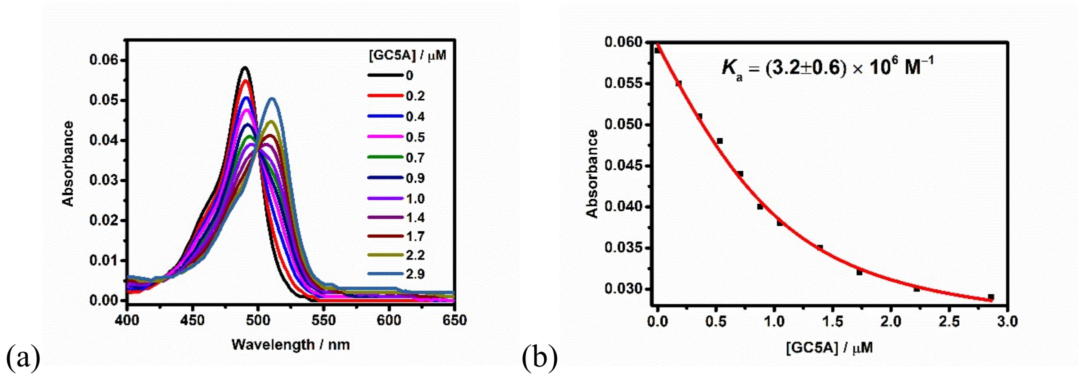

Figure S11. (a) Direct UV-Vis titration of Fl (1.0 μM) with GC5A (up to 2.9 μM). (b) The associated titration curve at λ = 490 nm was fitted according to a 1:1 binding stoichiometry.

3.5 Competitive fluorescence titration of phosphate and 6:0 LPA with GC5A

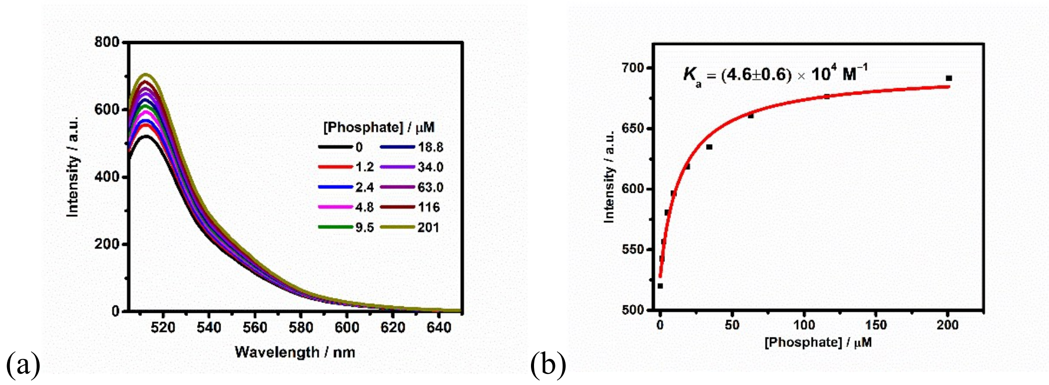

Figure S12. (a) Competitive fluorescence titration of GC5A•Fl (0.4/0.5 μM) with phosphate (up to 201 μM), λ_{ex} = 500 nm. (b) The associated titration curve at λ_{em} = 513 nm and fit according to a 1:1 competitive binding model.

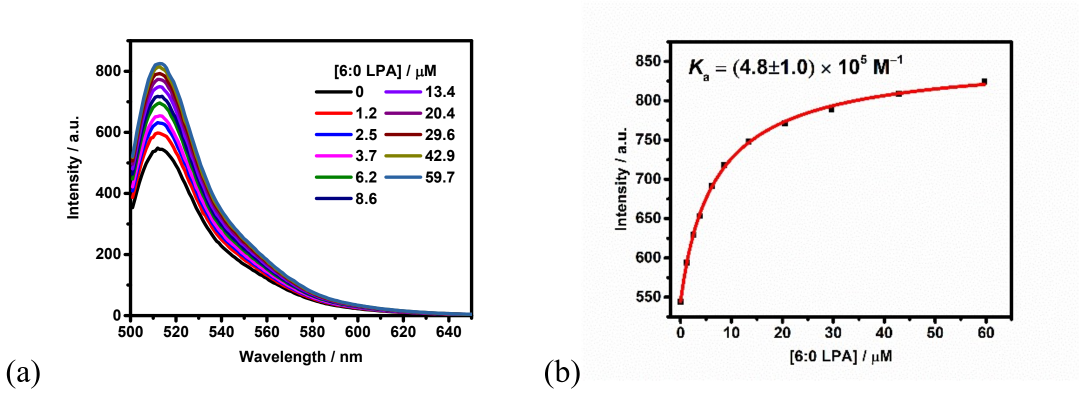

Figure S13. (a) Competitive fluorescence titration of GC5A•Fl (0.4/0.5 μM) with 6:0 LPA (up to 59.7 μM), λ_{ex} = 500 nm. (b) The associated titration curve at λ_{em} = 513 nm and fit according to a 1:1 competitive binding model.

3.6 Fluorescence, UV-Vis and DLS measurements of Fl with GC4A

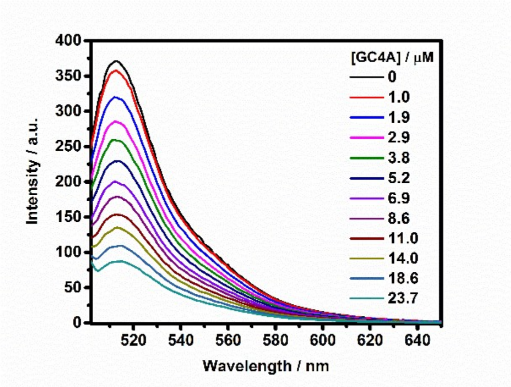

(a)

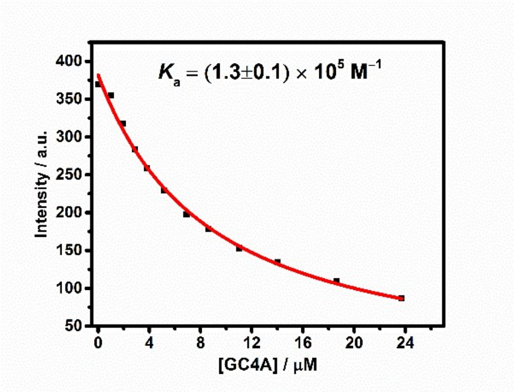

(b)

Figure S14. (a) Direct fluorescence titration of Fl (1.0 μM) with GC4A (up to 23.7 μM), λ_{ex} = 500 nm. (b) The associated titration curve at λ_{em} = 513 nm and fit according to a 1:1 binding stoichiometry.

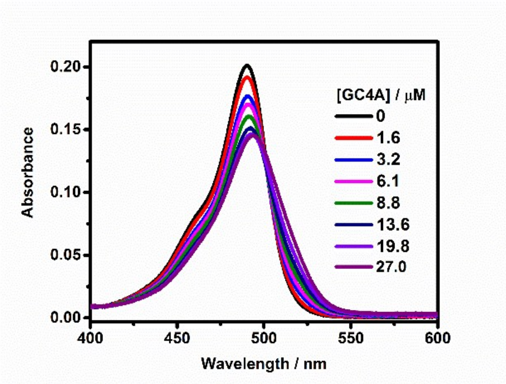

(a)

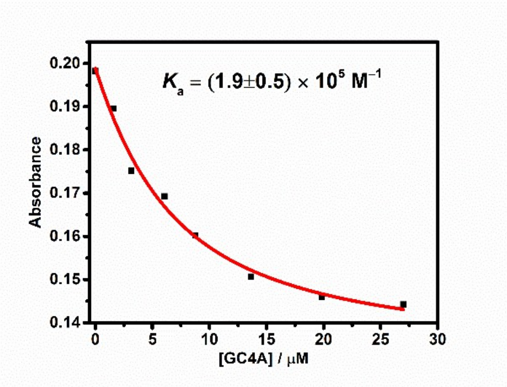

(b)

Figure S15. (a) Direct UV-Vis titration of Fl (3.0 μM) with GC4A (up to 27.0 μM). (b) The associated titration curve at λ = 490 nm and fit according to a 1:1 binding stoichiometry.

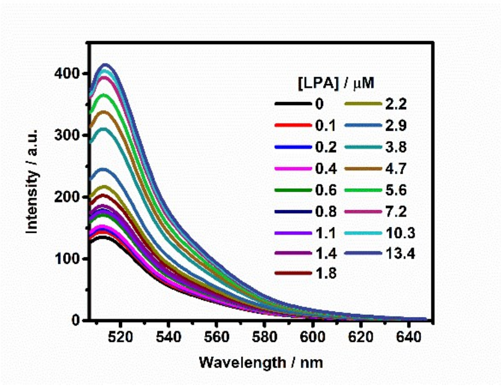

(a)

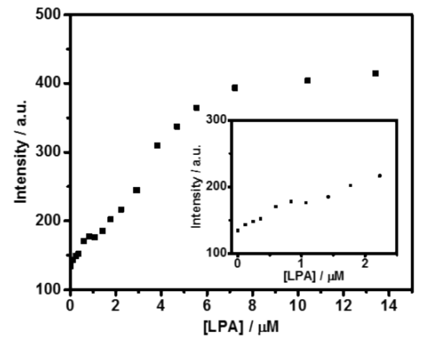

(b)

Figure S16. (a) Competitive fluorescence titration of GC4A•Fl (2.0/0.2 μM) with LPA (up to 13.4 μM), λ_{ex} = 500 nm. (b) The associated titration curve at λ_{em} = 513

nm and the enlarged figure at low range (inset). The complex curve cannot be fit to any simple 1:1, 1:2, or 2:1 binding isotherm.

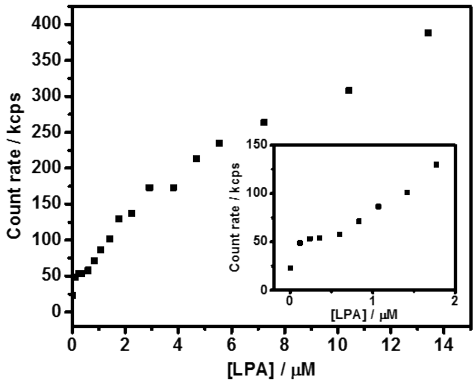

Figure S17. Scattering intensity of GC4A•Fl (2.0/0.2 μM) with different concentrations of LPA and the enlarged figure at low range (inset).

3.7 Fluorescence measurement of Fl with GC5A-CH_3

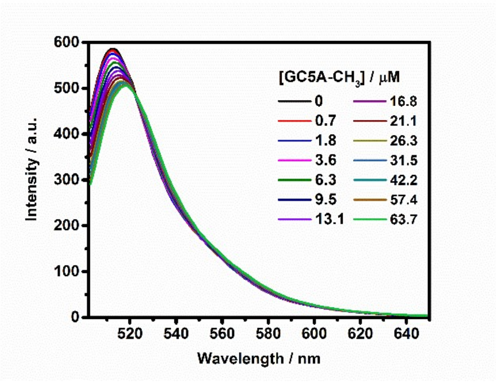

(a)

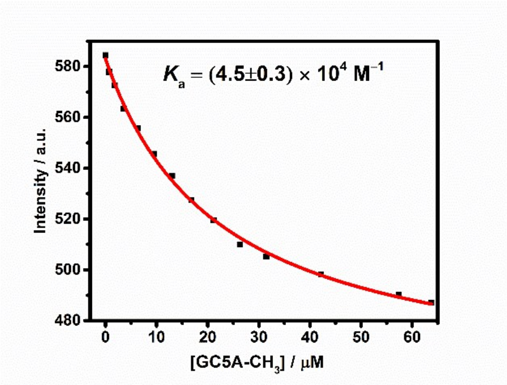

(b)

Figure S18. (a) Direct fluorescence titration of Fl (0.2 μM) with GC5A-CH_3 (up to 63.7 μM), λ_{ex} = 500 nm. (b) The associated titration curve at λ_{em} = 513 nm and fit according to a 1:1 binding stoichiometry. GC5A-CH_3 is flexible because the lower-rim methyl groups cannot restrict overturn of benzene ring units.

3.8 HMBC spectrum of 6:0 LPA

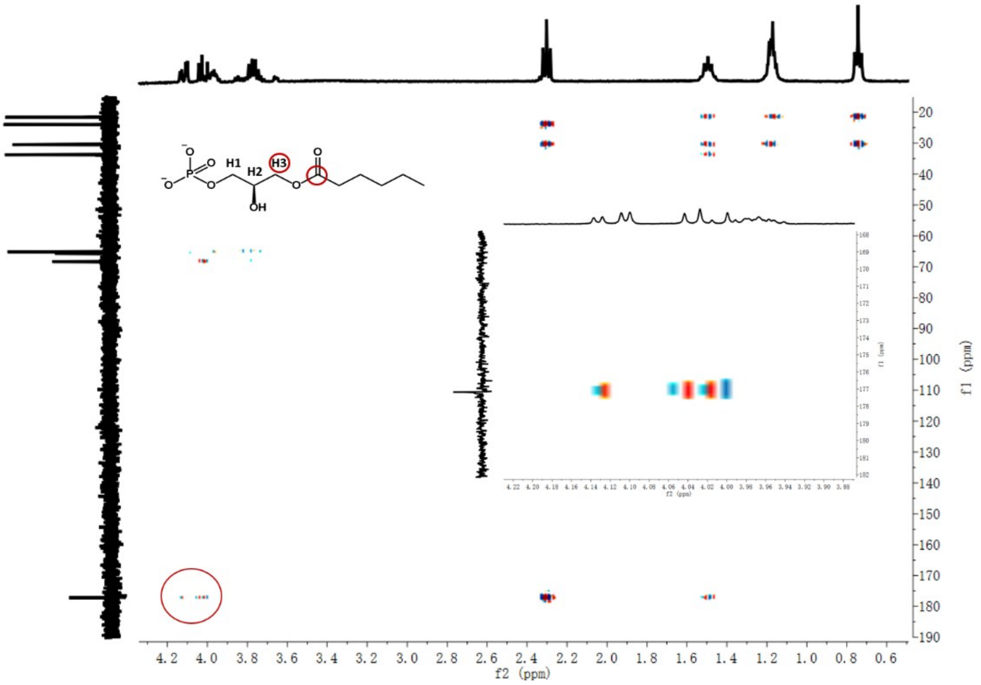

Figure S19. HMBC spectrum of 6:0 LPA showing correlations between ^{1}H and ^{13}C resonances, which was performed to assign the H3 peaks in ^{1}H NMR spectrum. Peaks at 4.00 ppm to 4.14 ppm from ^{1}H spectrum have correlations with peaks at 177.17 ppm from ^{13}C spectrum, which indicates they represent H3 protons.

3.9 ^{1}H NMR spectra of GC4A and 6:0 LPA

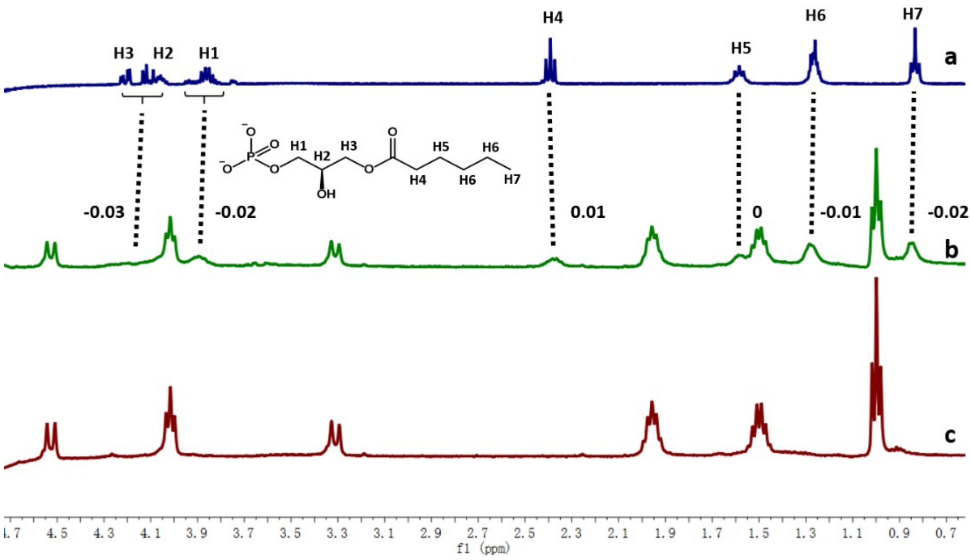

Figure S20. ^{1}H NMR spectra of (a) 6:0 LPA (1 mM), (b) 6:0 LPA (1 mM) with addition of GC4A (1 mM), and (c) GC4A (1 mM) in D_2O at 25 °C.

3.10 The limit of detection (LOD) for LPA in HEPES buffer

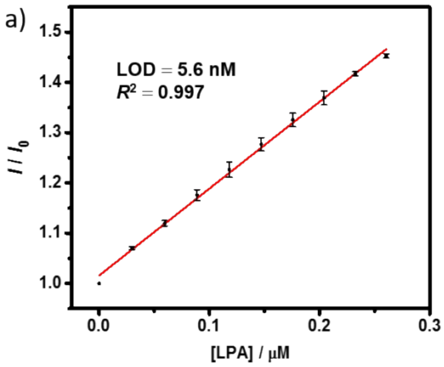

Figure S21. (a) Plot of I/I_0 against LPA concentration. I and I_0 are the fluorescence intensities of the GC5A•Fl (0.4/0.5 µM) reporter pair in the presence and absence of LPA (0–0.26 µM), λ_{ex} = 500 nm, λ_{em} = 513 nm, respectively. Error bars could not be shown if less than 0.005.

3.11 Fluorescence measurements of $AlPcS_4$ with GC5A and GC4A

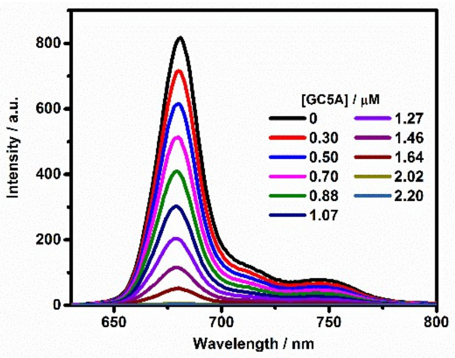

(a)

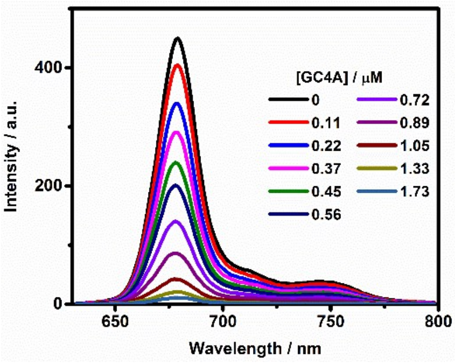

(b)

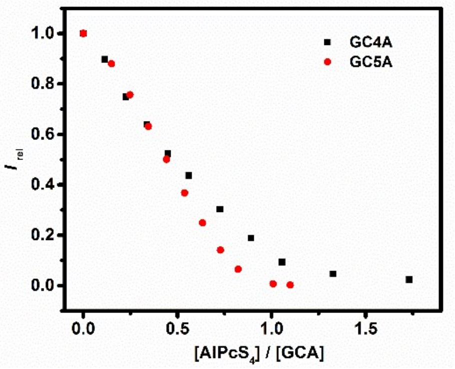

(c)

Figure S22. (a) Direct fluorescence titration of $AlPcS_4$ (2.0 μM) with GC5A (up to 2.20 μM), λ_{ex} = 608 nm. (b) Direct fluorescence titration of $AlPcS_4$ (1.0 μM) with GC4A (up to 1.73 μM), λ_{ex} = 608 nm. (c) The associated titration curves at λ_{em} = 680 nm, where strong binding accompanied with drastic fluorescence quenching of $AlPcS_4$ by GC4A and GC5A was observed.

3.12 Fluorescence responses of various biologically coexisting species with GC5A and GC4A

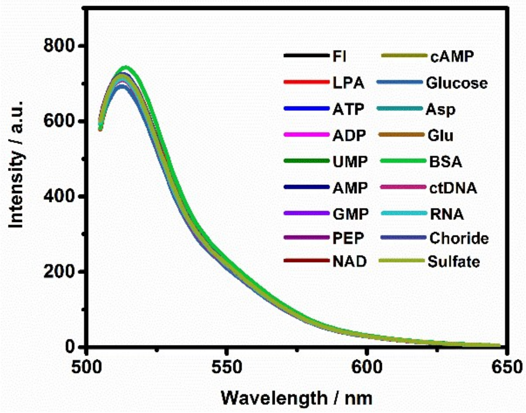

Figure S23. Fluorescence spectra of 0.5 μM Fl upon addition of various biologically coexisting species, λ_{ex} = 500 nm. All analytes exert no appreciable influence over the emission of Fl in the concentration range of the following titration experiments.

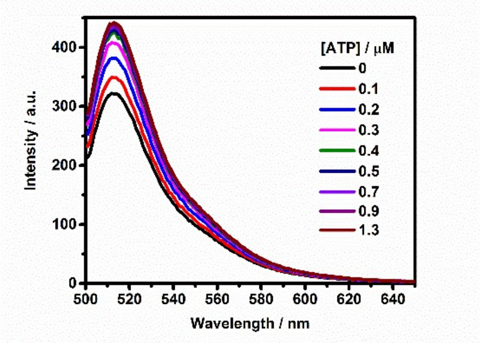

(a)

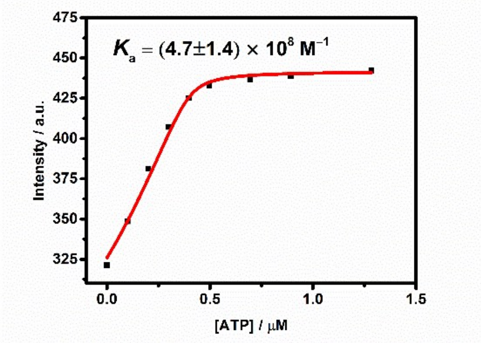

(b)

Figure S24. (a) Competitive fluorescence titration of GC5A•Fl (0.4/0.5 μM) with

ATP (up to 1.3 μM), λ_{ex} = 500 nm. (b) The associated titration curve at λ_{em} = 513 nm and fit according to a 1:1 competitive binding model.

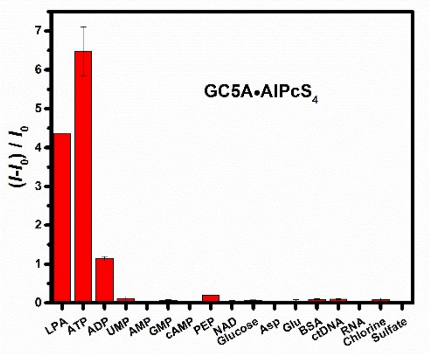

Figure S25. Fluorescence response of GC5A•AlPcS$_4$ (0.8/1.0 μM) at 680 nm (λ_{ex} = 608 nm) upon the addition of LPA and various biologically coexisting species (0.4 μM for small species and 0.15 mg/L for ctDNA, RNA and BSA) in HEPES buffer (10 mM, pH = 7.4) at 25 °C.

3.13 Analyzing cancerous and non-cancerous blood samples of mice by the GC5A•AlPcS$_4$ reporter pair

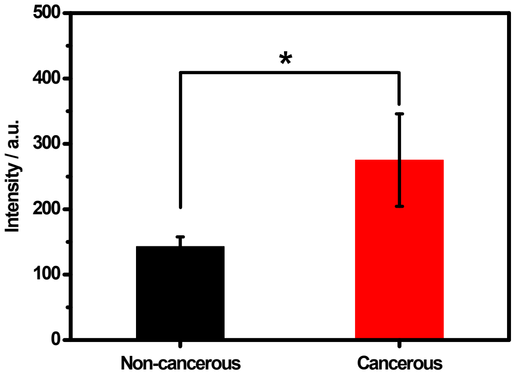

Figure S26. Fluorescence response of GC5A•AlPcS$_4$ (5.0/5.0 μM) at 680 nm (λ_{ex} = 608 nm) in tenfold diluted serum samples with water from healthy mice and mice with ovarian cancer at 25 °C. Significance was measured with Student's t test. *p < 0.05. Error bars represent the standard derivations of three independent studies.

4. Theoretical Calculations

All density functional theory (DFT) calculations were carried out using the Gaussion 09 program.[4] SMD solvation model[5] was used in all calculations. The geometry optimization was carried out using B3LYP/6-31G(d)[6] level of theory with Grimme's D3 dispersion correction,[7] which adopted the Becke−Johnson (BJ) damping function.[8] Default convergence criteria were used for the optimization with Gaussian 09. Atoms-in-molecules (AIM) and reduced density gradients (RDG) analysis were derived by using the Multiwfn software.[9] Molecular graphs were visualized by the

VMD program.[10]

The DFT-optimized structure of GC5A•6:0 LPA (Figure S27) displays one O−H···N (H···N distance 1.97 Å) and five N−H···O (average H···O distance 1.79 Å) intermolecular hydrogen bonds between guanidinium groups of GC5A and the phosphate head of 6:0 LPA.

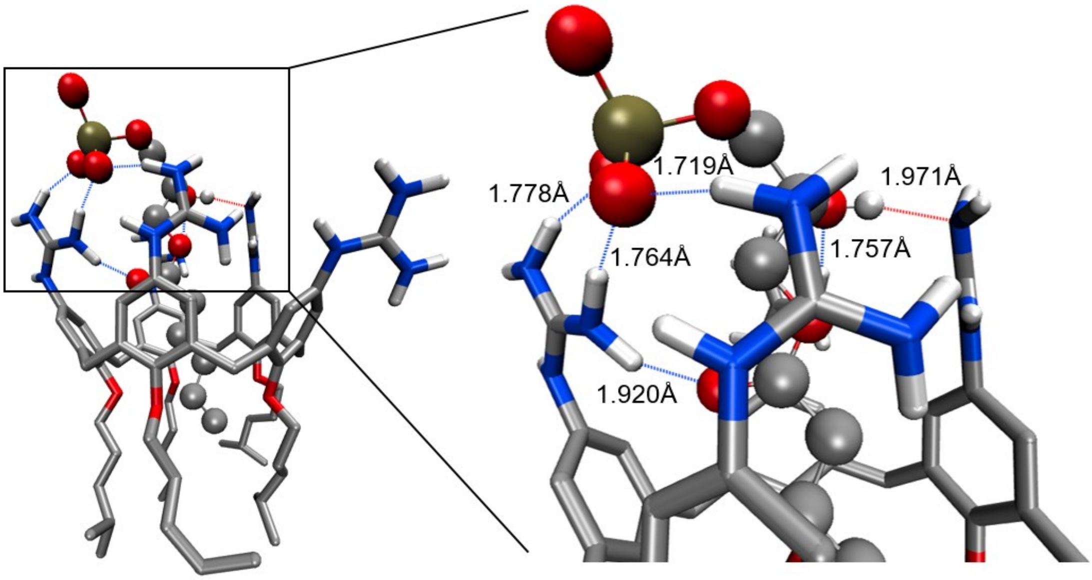

Figure S27. Optimized structure of the GC5A•6:0 LPA complex at the B3LYP-D3(BJ)/6-31G(d)/SMD(water) level of theory. Hydrogen atoms bonded to carbon are omitted for clarity. Evident hydrogen bonds are shown by red (O−H···N) and blue (N−H···O) dashed lines.

AIM analysis[11] was utilized for more detailed inspection of nature of the supramolecular interactions using the generated wave functions. In AIM analysis, the hydrogen bond interactions must satisfy the criterion[12] for the electron density at the bond critical points (BCP) in the range of 0.002−0.035 a.u., with the corresponding Laplacian ($\nabla^2\rho$) values being 0.024−0.139 a.u.. Intermolecular BCPs that satisfy the hydrogen bond criterion, associated with the corresponding bond paths in complex GC5A•6:0 LPA are shown in Figure S28. Table S1 shows properties at the BCPs: values of electron density (ρ), the Laplacian of electron density ($\nabla^2\rho$), the kinetic electron energy density (G), the potential electron energy density (V) and the total electron energy density (H). Furthermore, the energy of X−H···O (X=C, N, O) hydrogen bonds were estimated from the Espinosa correlation[13] ($E_{HB} = -0.5\times V$, where V is the potential electron energy density at BCP) (Table S1).

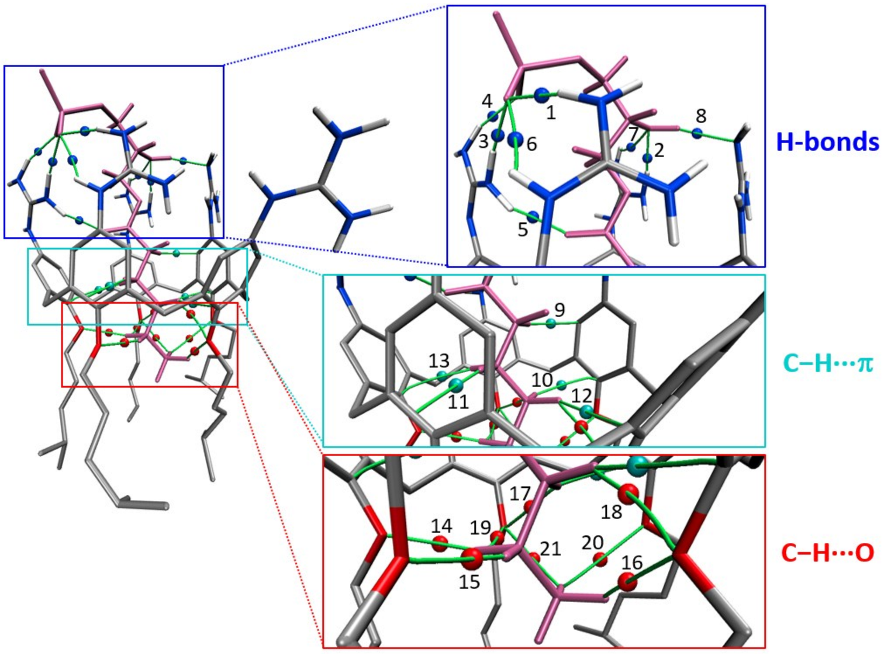

Figure S28. AIM topological analysis of weak interactions in the GC5A•6:0 LPA complex. The BCPs are shown as points.

The evident hydrogen bonds shown in Figure S27 were further validated by the AIM analysis results with bond energies between 29 and 53 kJ·mol^{-1} (Table S1). In addition, AIM analysis showed more interactions that satisfy the hydrogen bond criterion, which include C−H···π and C−H···O interactions (Figure S28 and Table S1). The delocalized π cloud of aromatic rings of GC5A and hydrogens at alkyl chain of 6:0 LPA bring about C−H···π interactions with the corresponding ρ_{bcp} values falling in the interval of 0.008−0.012 a.u. (Table S1). The values of ρ_{bcp} and its Laplacian indices are typical for weak hydrogen bonds, being larger in value than van der Waals (vdW) interactions. The C−H···O interactions between oxygen atoms at the lower rim of GC5A and hydrogens at the alkyl chain of 6:0 LPA (Figure S28) have hydrogen bond energy falling in the interval of 5−22 kJ·mol^{-1} (Table S1).

Table S1. AIM parameters for the nonbonding interactions in the GC5A•6:0 LPA complex. All quantities are expressed in a.u. except for E_{HB}. See text for definition of each of the quantities.

BCP	ρ	$\nabla^2\rho$	*G*	*V*	*H*	E_{HB}/kJ·mol^{-1}
1	0.047	0.137	0.037	-0.040	-0.003	52.707
2	0.043	0.128	0.034	-0.037	-0.002	48.343
3	0.042	0.125	0.034	-0.036	-0.002	47.435
4	0.040	0.119	0.032	-0.034	-0.002	44.578
5	0.026	0.092	0.023	-0.023	0.000	29.724
6	0.009	0.037	0.008	-0.007	0.001	9.730
7	0.006	0.026	0.005	-0.004	0.001	5.266
8	0.029	0.080	0.021	-0.022	-0.001	n.d.[a]
9	0.012	0.040	0.008	-0.007	0.001	n.d.[a]
10	0.012	0.042	0.009	-0.007	0.002	n.d.[a]
11	0.011	0.038	0.008	-0.006	0.002	n.d.[a]
12	0.009	0.034	0.007	-0.005	0.002	n.d.[a]
13	0.008	0.028	0.006	-0.004	0.001	n.d.[a]
14	0.020	0.065	0.016	-0.016	-0.000	21.532
15	0.014	0.045	0.011	-0.011	0.000	14.168
16	0.014	0.047	0.011	-0.010	0.001	13.688
17	0.010	0.038	0.008	-0.007	0.001	9.560
18	0.009	0.036	0.008	-0.006	0.001	8.146
19	0.007	0.028	0.006	-0.005	0.001	6.262
20	0.007	0.026	0.005	-0.004	0.001	5.558
21	0.006	0.025	0.005	-0.004	0.001	5.508

[a]These hydrogen bonds are not X−H···O (X=C, N, O) hydrogen bonds.

Weaker interactions such as vdW interaction cannot be analyzed by BCP within the AIM theory. Therefore, noncovalent interactions (NCI)[14] based on the relationship between the electron density ρ and the RDG were characterized to examine these interactions.

To probe the existence of weak interactions, 2D scatter graph was obtained by plotting the RDG versus electron density multiplied by the sign of the second Hessian eigenvalue (sign(λ_2)ρ) (Figure S29a). Attractive interactions are characterized by spikes at negative region of sign(λ_2)ρ, which is an indicator of interaction strength. The strong hydrogen bonds appear at the higher density values (sign (λ_2)ρ < −0.01 a.u.). Van der Waal interactions present at near zero sign(λ_2)ρ values region. Moreover, the 2D scatter graph was transformed into 3D graph by color mapping sign(λ_2)ρ to RDG isosurfaces to visualize noncovalent interactions (Figures S29b and S29c).

The RDG analysis results (Figure S29) also confirm the presence of hydrogen bond, C−H···π and C−H···O interactions in the AIM analysis. In addition, the RDG analysis sheds light on the existence of vdW interaction (green isosurfaces in the red circle in Figure S29c) between the alkyl chain of 6:0 LPA and alkyl chains of GC5A, which cannot be displayed by AIM analysis.

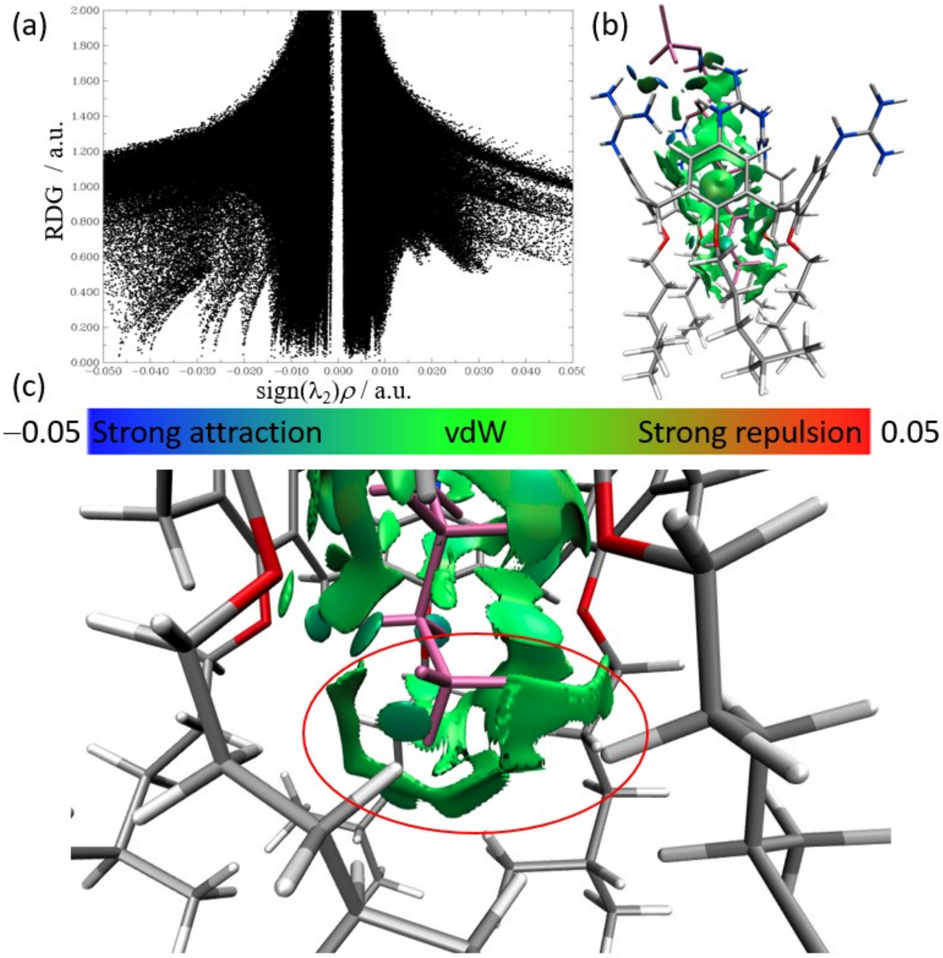

Figure S29. (a) Plots of the reduced density gradient versus the electron density multiplied by the sign of the second Hessian eigenvalue of the GC5A•6:0 LPA complex. Color-filled RDG isosurfaces (b and c) of the GC5A•6:0 LPA complex. Isovalue of RDG is set to 0.5.

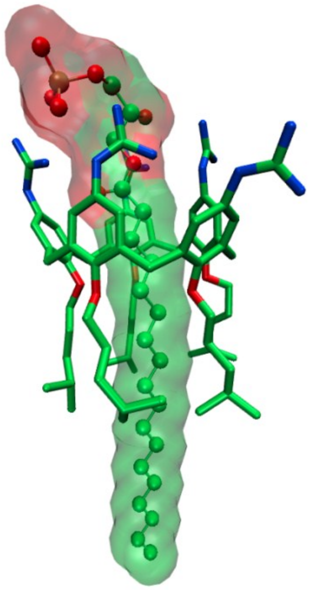

Figure S30. Optimized structure of the GC5A•18:0 LPA complex at the B3LYP-D3(BJ)/6-31G(d)/SMD(water) level of theory.

5. Reference

1. (a) T. Schrader, C. Blecking, W. Hu, R. Zadmard and A. Dasgupta, *Synthesis*, 2011, **08**, 1193-1204; (b) N. Moridi, C. Wackerlin, V. Rullaud, R. Schelldorfer, T. A. Jung and P. Shahgaldian, *Chem. Commun.*, 2013, **49**, 367-369; (c) F. Sansone, M. Dudic, G. Donofrio, C. Rivetti, L. Baldini, A. Casnati, S. Cellai and R. Ungaro, *J. Am. Chem. Soc.*, 2006, **128**, 14528-14536; (d) M. Mourer, R. E. Duval, C. Finance and J. B. Regnouf-de-Vains, *Bioorg. Med. Chem. Lett.*, 2006, **16**, 2960-2963.
2. (a) F. Arnaud-Neu, S. Fuangswasdi, A. Notti, S. Pappalardo and M. F. Parisi, *Angew. Chem., Int. Ed.*, 1998, **37**, 112-114; (b) D. Garozzo, G. Gattuso, A. Notti, A. Pappalardo, S. Pappalardo, M. F. Parisi, M. Perez and I. Pisagatti, *Angew. Chem., Int. Ed.*, 2005, **44**, 4892-4896.
3. G. Cafeo, G. Gattuso, F. H. Kohnke, A. Notti, S. Occhipinti, S. Pappalardo and M. F. Parisi, *Angew. Chem., Int. Ed.*, 2002, **41**, 2122-2126.
4. Gaussian 09, Revision E.01, M. J. Frisch, G. W. Trucks, H. B. Schlegel, G. E. Scuseria, M. A. Robb, J. R. Cheeseman, G. Scalmani, V. Barone, B. Mennucci, G. A. Petersson, H. Nakatsuji, M. Caricato, X. Li, H. P. Hratchian, A. F. Izmaylov, J. Bloino, G. Zheng, J. L. Sonnenberg, M. Hada, M. Ehara, K. Toyota, R. Fukuda, J. Hasegawa, M. Ishida, T. Nakajima, Y. Honda, O. Kitao, H. Nakai, T. Vreven, J. A. Montgomery, Jr., J. E. Peralta, F. Ogliaro, M. Bearpark, J. J. Heyd, E. Brothers, K. N. Kudin, V. N. Staroverov, T. Keith, R. Kobayashi, J. Normand, K. Raghavachari, A. Rendell, J. C. Burant, S. S. Iyengar, J. Tomasi, M. Cossi, N. Rega, J. M. Millam, M. Klene, J. E. Knox, J. B. Cross, V. Bakken, C. Adamo, J. Jaramillo, R. Gomperts, R. E. Stratmann, O. Yazyev, A. J. Austin, R. Cammi, C. Pomelli, J. W. Ochterski, R. L. Martin, K. Morokuma, V. G. Zakrzewski, G. A. Voth, P. Salvador, J. J. Dannenberg, S. Dapprich, A. D. Daniels, O. Farkas, J. B. Foresman, J. V. Ortiz, J. Cioslowski, and D. J. Fox, Gaussian, Inc., Wallingford CT, 2013.
5. A. V. Marenich, C. J. Cramer and D. G. Truhlar, *J. Phys. Chem. B*, 2009, **113**, 6378-6396.
6. P. J. Stephens, F. J. Devlin, C. F. Chabalowski and M. J. Frisch, *J. Phys. Chem.*, 1994, **98**, 11623-

11627.
7. S. Grimme, J. Antony, S. Ehrlich and H. Krieg, *J. Chem. Phys.*, 2010, **132**, 154104.
8. S. Grimme, S. Ehrlich and L. Goerigk, *J. Comput. Chem.*, 2011, **32**, 1456-1465.
9. T. Lu and F. Chen, *J. Comput. Chem.*, 2012, **33**, 580-592.
10. W. Humphrey, A. Dalke and K. Schulten, *J. Mol. Graphics*, 1996, **14**, 33-38.
11. R. F. W. Bader, *Chem. Rev.*, 1991, **91**, 893-928.
12. U. Koch and P. L. A. Popelier, *J. Phys. Chem.*, 1995, **99**, 9747-9754.
13. E. Espinosa, E. Molins and C. Lecomte, *Chem. Phys. Lett.*, 1998, **285**, 170-173.
14. E. R. Johnson, S. Keinan, P. Mori-Sanchez, J. Contreras-Garcia, A. J. Cohen and W. Yang, *J. Am. Chem. Soc.*, 2010, **132**, 6498-6506.
